# Conditions for invasion and persistence of a satellite that does not affect the replication and virulence of its helper virus

**DOI:** 10.1093/ve/veag063

**Published:** 2026-09-24

**Authors:** Rafael de Andrés-Torán, Bruno Cuevas-Zuviría, Mark P Zwart, Miguel Ángel Mora, Aurora Fraile, Fernando García-Arenal

**Affiliations:** Centro de Biotecnología y Genómica de Plantas (CBGP UPM-CSIC/INIA), Universidad Politécnica de Madrid and E.T.S.I. Agronómica, Alimentaria y de Biosistemas, Campus de Montegancedo, UPM, 28223 Pozuelo de Alarcón, Madrid, Spain; Centro de Biotecnología y Genómica de Plantas (CBGP UPM-CSIC/INIA), Universidad Politécnica de Madrid and E.T.S.I. Agronómica, Alimentaria y de Biosistemas, Campus de Montegancedo, UPM, 28223 Pozuelo de Alarcón, Madrid, Spain; Netherlands Institute of Ecology (NIOO-KNAW), Droevendaalsesteeg 10, Wageningen 6708PB, The Netherlands; Centro de Biotecnología y Genómica de Plantas (CBGP UPM-CSIC/INIA), Universidad Politécnica de Madrid and E.T.S.I. Agronómica, Alimentaria y de Biosistemas, Campus de Montegancedo, UPM, 28223 Pozuelo de Alarcón, Madrid, Spain; Centro de Biotecnología y Genómica de Plantas (CBGP UPM-CSIC/INIA), Universidad Politécnica de Madrid and E.T.S.I. Agronómica, Alimentaria y de Biosistemas, Campus de Montegancedo, UPM, 28223 Pozuelo de Alarcón, Madrid, Spain; Centro de Biotecnología y Genómica de Plantas (CBGP UPM-CSIC/INIA), Universidad Politécnica de Madrid and E.T.S.I. Agronómica, Alimentaria y de Biosistemas, Campus de Montegancedo, UPM, 28223 Pozuelo de Alarcón, Madrid, Spain

**Keywords:** satellite tobacco mosaic virus, tobacco mild green mosaic virus, transmission, infection dynamics, invasion, persistence

## Abstract

Satellites are subviral agents that depend on a helper virus for functions essential to complete their infection cycle, while the helper virus may infect and be transmitted in the absence of the satellite. Thus, satellites are cheats that compete with the helper virus for its resources, and may modulate its replication, infection dynamics, evolution, and the coexistence among viruses. Understanding the conditions for satellite invasion and persistence is therefore relevant to understanding both the prevalence of single viruses and virus community dynamics. This is the goal of this study, centred in satellite tobacco mosaic virus (STMV), and its helper virus tobacco mild green mosaic virus (TMGMV). We use epidemiological data on STMV occurrence and infection dynamics in TMGMV-infected *Nicotiana glauca* populations, and experimentally estimate relevant parameters to feed models that predict STMV invasion and persistence conditions. We show that under field conditions, STMV has no significant effects on TMGMV accumulation or pathogenesis in *N. glauca*. However, the presence of STMV significantly reduces the horizontal transmission rate of TMGMV, and we showed experimentally that STMV transmission occurs in only a fraction of TMGMV transmission events. Model simulations showed that a requirement for STMV invasion at realistic transmission rates was a low virulence, a condition met as shown by previous studies of the virulence of TMGMV in *N. glauca*. Simulations that considered variation in the density of the host plant population identified a high transmission rate as a condition for the persistence of STMV, which disappears from the TMGMV population at low plant densities. A major conclusion is that STMV interferes with TMGMV transmission despite not interfering with TMGMV accumulation. Thus, conditions favouring a high transmission rate are required for invasion and persistence of STMV, similarly to satellites that interfere with helper virus replication, suggesting these conditions may be general for other satellite-helper virus systems.

## Introduction

Satellites are subviral agents that depend on a helper virus for essential functions of their infection cycle, such as replication, colonization of the infected host, or transmission and that, at odds with defective or defective interfering genomes (DI), have little or no sequence homology with their helper or other viruses (Palukaitis 2016). Some satellites (satellite viruses) encode the coat protein that forms their own particles and depend on the helper virus for replication, while most (satellite nucleic acids) do not and depend on the helper virus also for encapsidation. The interaction between satellite and helper virus is asymmetric, as while the satellite depends on the helper virus to complete its infection cycle, the helper virus may infect and be transmitted in the absence of the satellite. Thus, satellites are cheats, as are DIs, but while DIs typically go extinct within the course of an individual infection, satellites may be efficiently transmitted among hosts and persist in the helper virus population for long times (Celix et al. 1997, Nee 2000, Leeks et al. 2023). As satellites compete with the helper virus for its resources they may modulate its replication, pathogenesis and virulence (Palukaitis 2016, Gnanasekaran and Chakraborty 2018), and it is predicted that satellites will impact on the helper virus infection dynamics and evolution (Zhang et al. 2000, Alizon et al. 2013, Sofonea et al. 2015, Alizon 2023, Leeks et al. 2023, Lázaro et al. 2024), the coexistence among viruses (Nee 2000, Lucia-Sanz et al. 2022, Lázaro et al. 2024) and, hence, may affect virus community assembly and diversity (Norberg et al. 2023). The conditions for satellite invasion and persistence are therefore highly relevant to understanding the prevalence of single viruses as well as virus community dynamics.

Most reported satellites are associated to plant viruses. There is abundant evidence for satellite persistence in populations of ribonucleic acid (RNA) and desoxyribonucleic acid (DNA) plant viruses, where they are often associated with epidemics of specific diseases such as tomato necrosis (Jordá et al. 1992, Grieco et al. 1997), Saint Augustine grass decline (Cabrera and Scholthof 1999); *Ageratum* yellow vein disease (Saunders et al. 2000), or cotton leaf curl (Briddon 2003). Much effort has been made to understand the molecular bases of satellite—helper virus dependency and of the modulation of the helper virus replication and pathogenicity (Zhou 2013, Fiallo-Olivé et al. 2016, Gnanasekaran and Chakraborty 2018, Yang et al. 2019, Wrzesińska-Krupa and Obrępalska-Stęplowska 2025). In contrast, little attention has been paid to the conditions in which satellites can invade and persist in the helper virus population and to the effects on the helper virus infection dynamics and evolution. The few studies on these topics have focused on satellites that have a severe effect on the pathogenesis and accumulation of the helper virus. In fact, field evidence for satellite invasion and extinction has been reported only, to our knowledge, for the satellite RNA (CMVsatRNA) of cucumber mosaic virus (CMV, *Cucumovirus CMV*, *Bromoviridae*) (Jordá et al. 1992, Alonso-Prados et al. 1998), and field data were the basis for experimental and model analysis of virulence evolution and of the invasion and persistence conditions (Escriu et al. 2000, Betancourt et al. 2013, 2016). Here we examine the occurrence, analyse infection dynamics, and identify invasion and persistence conditions of a satellite that has little or no effect on the multiplication and pathogenesis of its helper virus, the satellite tobacco mosaic virus (STMV).

STMV (*Virtovirus tabaci*, *Tomosoviridae*) was first reported in association with tobacco mild green mosaic virus (TMGMV, *Tobamovirus mititessellati*, *Virgaviridae*) in infected *Nicotiana glauca* Grah. (Solanaceae) plants from California (Valverde and Dodds 1986). STMV has 17 nm diameter spherical particles with T = 1 symmetry that encapsidate a 5′ capped 1 058 nucleotides (nt) messenger-sense single-stranded RNA genome that encodes a coat protein (Mirkov et al. 1989). STMV is widespread in TMGMV-infected *N. glauca* throughout California (Valverde 1991). Although in nature STMV has only been reported associated with TMGMV in *N. glauca*, its replication and within-plant movement are supported by other tobamoviruses and in other solanaceous hosts such as tobacco, pepper and tomato (Dodds 1998). The effects of STMV on TMGMV are host-dependent; for example, in certain pepper cultivars STMV is associated with the attenuation of TMGMV-induced symptoms and the accumulation of the helper virus is greatly suppressed (Dodds 1998). However, in *N. glauca* only a slight negative effect on the accumulation of TMGMV has been reported, and its presence does not modify the pathogenicity of TMGMV (Valverde 1991, Dodds 1998). The helper virus, TMGMV, was first isolated from *N. glauca* plants from the Canary Islands (Mckinney 1929), and later has been reported infecting this host plant in many regions of the world (Randles et al. 1981, Fraile et al. 1996). As other tobamoviruses, TMGMV is vertically transmitted through the seed, and horizontally by plant-to-plant contact and, probably, by insect pollinators. TMGMV strains show variation in the presence or absence of a duplication in the 3′ untranslated region (3’UTR) of the genome (Long-3’UTR and Short-3’UTR isolates) (Bodaghi et al. 2004, Bera et al. 2018). In *N. glauca*, Long-3’UTR and Short-3’UTR isolates differ in important life-history traits, such as the kinetics of multiplication, virulence, and rates of vertical transmission (de Andrés-Torán et al. 2025). *Nicotiana glauca* is native to the central eastern Andes in South America, but presently it is widely distributed in semi-arid regions of the world, including southeastern (SE) Spain, where it was introduced in the first half of the XIX Century (MITECO 2020). *Nicotiana glauca* is an evergreen bush that can reach between 3 and 7 m of height and live up to 10 years (MITECO 2020, de Andrés-Torán et al. 2025). In the Mediterranean region, *N. glauca* is pollinated by hymenopteran and lepidopteran insects (Ollerton et al. 2012, MITECO 2020) that may contribute to the horizontal transmission of viruses. *Nicotiana glauca* is a colonizer species with a poor competitive potential and is thus found in disturbed habitats such as roadsides or gullies that are occasionally flooded. In SE Spain, it has a patchy distribution in stands of 20–200 individuals, where TMGMV incidence is of about 70% (Rodríguez-Cerezo et al. 1991, de Andrés-Torán et al. 2025).

In this study, we report the presence on STMV in TMGMV-infected *N. glauca* plants in SE Spain, and analyse its incidence across space and time. We then experimentally estimate relevant epidemiological parameters to feed mean-field models to predict STMV invasion conditions. We test the hypothesis that effects of satellites on helper virus replication will determine conditions for satellite—helper virus coexistence.

## Material and methods

### Study area and field data collection

Field work was performed in February 2018 and February 2020 in the province of Almería, SE Spain. Collections were conducted at six *N. glauca* populations on a 60 km East–West transect (de Andrés-Torán et al. 2025). Population names and coordinates are: San José (36°46′07.4”N2°06′34.5”W), La Datilera (36°48′40.8”N 2°08′32.2”W), Rambla Morales (36°48′29.7”N2°13′57.3”W), Rambla Amoladeras (36°49′44.4”N 2°16′07.6”W), AL3115 (36°50′31.2”N2°17′47.8”W), and El Edar (36°51′13.5”N 2°20′28.3”W). Plants were sampled at fixed distances on predetermined itineraries covering the extent of each population, regardless of plants showing symptoms of virus infection or not. All plants encountered along the itineraries were sampled in the smaller populations, and one out of three plants in the larger ones. The population extent and census varied from ~ 320 m^2^ and 20 plants (El Edar) to ~ 2000 m^2^ and 100–350 plants (San José, La Datilera). Samples consisted of four to six leaves from different branches if the plant had more than one, and symptoms were recorded for each sampled plant. Each collection of leaves from an individual plant was placed in a plastic bag that was sealed and transported on ice to the laboratory, where leaf sap was extracted and the remaining material was stored at −80°C. Field sampling was also done at a different site in the province of Almería, Talud de Carboneras (36° 57’ N, 2° 10’ W), at different times between February 1992 and January 2003 (February 1992, November 1992, April 1994, January 1996, May 1999, January 2003). This was a slope running East–West generated by the construction of a road in 1991. The newly generated slope was colonized by *N. glauca* plants by 1992, and after 2003 *N. glauca* plants were totally displaced by the native xerophytic bushland of the region. The extent of the slope was ~ 2750 m^2^ and it was divided into 55 10 × 3 m slots. At each visit, *N. glauca* plant density and coverage at each slot were estimated visually, and leaves were sampled from one randomly chosen plant per slot and processed as described above.

### Virus detection, quantification and nucleotide sequence determination

Total RNA was extracted from leaves of plants collected in the field or from experiments using a TRIzol-based procedure with the NZYol reagent (NZYtech, Lisbon, Portugal) following the manufacturer’s instructions, after grinding 0.1 g of leaf tissue in liquid nitrogen. The presence of TMGMV or STMV RNA in total RNA extracts from leaves was analysed by reverse transcription (RT) and polymerase chain reaction (PCR) (RT-PCR) using first-strand cDNA synthesis kit (NZYtech, Lisbon, Portugal) and Phusion polymerase (Thermo Fisher Scientific, MA, USA), with specific primers that amplify the coat protein gene and the 3’UTR of TMGMV (TMGMVuRNA3, identical to nucleotide (nt) positions 5562–5590 of TMGMV M34077, and CGM3, complementary to the 3′ end positions 6338–6355 of TMGMV M34077) (Bera et al. 2018), or a fragment of the coat protein gene of STMV (primers STMV-136-F and STMV-136-R, complementary or identical, respectively, to nt positions 497–524 and 610–663 of STMV M25782). Amplicons were analysed by agarose gel electrophoresis. Quantification of TMGMV titre was performed by quantitative RT-PCR (RT-qPCR) using primers complementary or identical to positions 5969–5990 and 6037–6058 of the TMGMV genome (accession no. M34077) (Fraile et al. 2014). For each sample, 100 ng of total RNA were utilized with Brilliant III Ultra-Fast SYBR Green QRT-PCR Master Mix (Agilent Technologies, CA, USA) according to the manufacturer’s recommendations in a final volume of 10 μl. Assays were performed in duplicate on a LightCycler 480 II real-time PCR system (Roche, IN, USA). Levels of viral RNA were deduced from comparison with standard curves produced using a set of serial dilutions in water of purified virion RNA. For the determination of the nucleotide sequence of the coat protein gene of STMV, amplicons were obtained by RT-PCR on total RNA extracts from leaves of infected plants using primers complementary and identical to nucleotide positions 138–159 and 643–664 of STMV genome (Accession No. M25782.1). Sanger sequencing was outsourced to Stab Vida (Lisbon, Portugal).

### Experimental estimation of rates of horizontal transmission of TMGMV and STMV

TMGMV isolates to be used in the same experiment were multiplied simultaneously in the susceptible host *Nicotiana tabacum* L cv. Samsun and viral particles were purified as in (Bruening et al. 1976). Three field isolates of TMGMV, one of a Long-3’UTR type, one of a Short-3’UTR type, and one of a Long-3’UTR type maintaining a STMV were used. For the transmission experiment, *N. tabacum* cv. Samsun plants were grown separately in 15 cm diameter, 1.5 l pots at 24°C and 16 h light photoperiod in a greenhouse. When plants had fully expanded the first two true leaves (about 3 weeks after sowing), they were transplanted to 1 m plastic throughs, randomly distributed and physically separate by at least 1 m in the greenhouse, filled with soil and arranged in rows of 10 plants separated by 5 cm. The first plant of each row of 10, was inoculated with 400 ng of virus particles in 0.1 M Na_2_HPO_4_ buffer, pH 7.2, ensuring that transmission occurred by contact between leaves of adjacent plants, as the experiments was carried in an insect-proof greenhouse where no pollinators or other insects were present during the experiment. The experiment involved four rows per isolate taken as replicates, and the progress of infection along each row was monitored daily by the appearance of symptoms. As symptom expression can be delayed relative to infection, the infection status of each plant was also checked daily by retroinoculation in *N. tabacum* cv. Xanthi-nc, a local lesion host where necrotic local lesions (nll) developed if the virus was present. This is an easy and reliable way to detect infection. TMGMV accumulation was quantified in the inoculated leaves of the inoculated plants at 7 days post inoculation (dpi), and in systemically infected leaves at seven and 12 dpi. At the end of the experiment, three 5 mm diameter disks from three systemically infected leaves were also collected from all the plants of the experiment to confirm the presence of TMGMV and STMV by RT-PCR. This assay allowed detecting infection by TMGMV and STMV separately, which the nll did not allow. During the experiment plants were kept at the above greenhouse conditions.

To estimate the rate of transmission of STMV per TMGMV transmission event, total RNA extracts from leaves of five field *N. glauca* plants infected by TMGMV plus STMV (Ng 94/5, Ng 94/10, Ng 96/22 and Ng 03/5, with a Long-3’UTR TMGMV infection, and Ng 92/39 with a Short-3’UTR TMGMV infection) were used to inoculate *N. tabacum* cv. Xanthi-nc fully-expanded leaves in a greenhouse. Each nll indicates a successful infection at a different infection site, that is, a different transmission event. Thus, the nll assay allowed estimating the relative infectivity of STMV and TMGMV across isolates by counting the number of nll in which STMV was present. Such counting was done in two complementary ways, as follows. At five dpi, 40 nll resulting from the inoculation of each field plant RNA were harvested. In 20 of these nll, total RNA was extracted, and the presence of STMV analysed by RT-PCR. The other twenty nll were used to mechanically inoculate one plant per nll of *N. tabacum* cv. Samsun, and at 14 dpi, tissue samples from three different upper leaves were collected, RNA was extracted and the presence of TMGMV and STMV analysed by RT-PCR.

### Epidemiological model

The dynamics of TMGMV and STMV infection in *N. glauca* populations was analysed by the mean field model described by the ordinary differential equations (ODEs) given in Equation 1.


(1) \begin{align*} & \dot{S}=\theta (t)-{\beta}_I SI-{\beta}_D SD- mS\nonumber\\{}& \dot{I}={\beta}_I SI-\gamma{\beta}_D ID+\left(1-\gamma \right){\beta}_D SD-\left(m+\alpha \right)I \\{}& \nonumber\dot{D}=\gamma{\beta}_D SD+\gamma{\beta}_D ID-\left(m+\alpha \right)D \end{align*}

Plants belong to one of three compartments: susceptible non-infected (*S*), infected by TMGMV (*I*) or infected by TMGMV+STMV (*D*). Susceptible hosts enter and exit the population through natural birth (*θ*) and mortality (*m*) parameters such that the population is constant at a steady unit value (*S+I+D=1*). *S* plants are infected by TMGMV and STMV from *I* and *D* plants through mass action terms multiplied by specific *transmission rate* parameters (${\beta}_I$ and ${\beta}_D$), and STMV can also be transmitted from *D* to *I* plants. The transmission rate of STMV is modified by parameter *γ*, as STMV is not transmitted in all TMGMV+STMV transmission events, such that *S-D* contacts may result in *I* plants. Infection by both TMGMV and TMGMV+STMV modifies the mortality rate by a virulence parameter *α* that is the same for *I* and *D* plants, as STMV infection does not modify TMGMV titre or symptoms (see Results). For simplicity, we considered that infected plants are instantly infectious, that is, there is no latent period. The natural mortality parameter was set at the fixed value of *m* = 0.042 month^−1^, considering the average life span of *N. glauca* plants to be of about 2 years (de Andrés-Torán et al. 2025). Parameters β and γ were estimated experimentally (see details in the Results section). Stationary state solutions were mathematically derived by solving the scenarios in which $\dot{S}=\dot{I}=\dot{D}=0$.

Model trajectories were computed using the JAX ODE solving package. Parameters in the model and their values in simulations are indicated in Table 1. Initial conditions were ${I}_0$ = 0.006 and ${D}_0$ = 0.004 to reproduce the observed relative prevalence of TMGMV and STMV. The stationary states were computed by simulating trajectories for long periods of time (t > 1000 months). To infer the ratio between STMV and TMGMV transmission rates, Approximate Bayesian Computation (ABC) was employed using the Numpyro package (Phan et al. 2019). The NUTS algorithm was employed to sample 1000 samples in four different chains after 500 initial burn-in steps.

**Table 1:** Description and values of parameters used in epidemiological models.

***Parameter***	***Description (units)***	***Value***
*m*	*Per capita mortality rate of uninfected plants (month^−1^)*	*0.042*
*α*	*Per capita increase in mortality rate due to infection (month^−1^)*	*From −0.042 to 0.2*
*β_I_*	*Per capita rate of transmission of pathogen i (month^−1^)*	*From 0.0 to 1.0*
*δ*	*Ratio between per capita transmission rates from plants infected by TMGMV + SMTV (β_D_) and plants infected by TMGMV (β_I_) (unitless)*	*0.8*
*γ*	*Probability of satellite transmission per transmission event (unitless)*	*0.42*

### Models for estimating the effect of STMV on TMGMV rates of horizontal transmission

To determine the ratio between the *per capita* transmission rates from plants infected by TMGMV+SMTV and plants infected by TMGMV ($\delta; {\beta}_D=\delta{\beta}_I$) two different models were fitted to the experimental data obtained as described under the above heading ‘Experimental estimation of rates of horizontal transmission of TMGMV and STMV’.

First, we used a model derived from Equation 1, in which natural mortality and virulence were not considered, as no plants died during the experiment. Transmission rates were multiplied by a transmission decay term (${e}^{-{\beta}_I\epsilon t}$) to account for an age-related decrease in plant susceptibility, as suggested by the experiment results. The transmission decay term includes ${\beta}_I$ to avoid collinearity between transmission decay and transmission rate, and the term *ε* determines the ratio of the decay of transmission compared to ${\beta}_I$. Finally, because experimental data are based on nll assays that do not allow detection of STMV infection, the model was fitted to the total infected plants, with no distinction of infection by TMGMV or by TMGMV+STMV. The model is described by Equation 2:


(2) \begin{align*} \dot{S}& =-{\beta}_I{e}^{-{\beta}_I\epsilon t}\left( SI+\delta SD\right)\nonumber\\{}\dot{I}& ={\beta}_I{e}^{-{\beta}_I\epsilon t}\left( SI+\left(1-\gamma \right)\delta SD-\gamma \delta DI\right)\\{}\dot{D}& =\gamma{\beta}_I\delta{e}^{-{\beta}_I\epsilon t}\left( SD+ DI\right) \nonumber \end{align*}

Second, we used a simpler model (Equation 3) which collapses the dynamics of TMGMV- and TMGMV+STMV-infected plants into a single compartment and assumes prevalence-independent transmission.


(3) \begin{align*} \dot{I}&=\beta{e}^{-\beta \epsilon t}\nonumber\\{}\dot{D}&=\delta \beta{e}^{-\beta \epsilon t} \end{align*}

Both models were fitted to the data using ABC in NumPyro (Phan et al. 2019). The NUTS algorithm was employed to sample 1000 Markov Chain Monte Carlo (MCMC) samples in four different chains after 500 initial warm-up steps. The population compartment values at the measured points were computed by solving the differential equations with the specific initial conditions for each treatment, which consisted of either TMGMV-only (*I*) inoculum or TMGMV+STMV (*D*) inoculum. We assumed that observation noise follows a *Beta* distribution, with a *σ* hyper-parameter to control the level of noise. Uninformative prior distributions were assigned to unknown parameters $\sigma, {\beta}_I$, *δ* and *ε*. Only *δ* values were retained, while all other parameters were discarded after fitting.

### Data analysis

Data on the number of plants infected or symptomatic in the field were analysed by contingency tests. Data on the rate of change of virus incidence in the field was analysed through a logistic regression of the disease progress curve, allowing the calculation of the rate of growth (infection rate, *r*_max_) of the incidence over time (Campbell and Madden 1990). Data on virus accumulation in field plant samples, or in experiments, were analysed similarly: Outliers were removed using Grubbs’ test and the distribution that best fitted the data was evaluated by means of Shapiro–Wilk test. Homogeneity of variance was analysed with a Levene’s test. After this, full factorial generalized linear models (GLMs) with different factors were performed. Data on the progress of infection through horizontal transmission of TMGMV was analysed by the area under the disease-progress curve (AUDPC) per line of plants. AUDPC values were calculated by reconstructing the disease progress curve from the time of appearance of nll as the result of retroinoculation from individual plants in the nll host. At each observation date, cumulative disease incidence was calculated as the proportion of plants that had developed lesions, and AUDPC was obtained by trapezoidal integration of cumulative incidence over time. Distribution and homogeneity of variance of AUDPC values were analysed as described above, and AUDPC values between the different treatments were compared by means of an analysis of variance (ANOVA). All statistical analyses were performed using R (4.3.1) or IBM SPSS Statistics Version 29.0.0.0 (241).

## Results

### Prevalence of STMV in *N. glauca* populations and effects on TMGMV accumulation

Detection of STMV was performed in TMGMV-infected *N. glauca* plants by RT-PCR with specific primers. The identity of STMV was confirmed by nucleotide sequence determination of the coat protein gene and phylogenetic analyses that showed that sequences from *N. glauca* plants from SE Spain were closely related to that of a reference STMV from California, USA (Fig. S1). Detections from the six *N. glauca* populations sampled in February 2018 and February 2020 (Table 2 and Table S1) showed that STMV occurred in the six *N. glauca* populations, with a prevalence over TMGMV-infected plants, aggregated over populations and collections, of 0.56. STMV infection was not associated with 3’UTR size (${\chi}_1^2$ = 2.430, *P* = .119). STMV did not seem to affect the prevalence of TMGMV within a population, which was similar without or with the satellite virus (0.21 [0.05–0.38] and 0.27 [0.08–0.42], respectively). Also, STMV did not affect symptom expression; the number of plants infected by TMGMV or TMGMV + STMV that showed mosaics being similar (${\chi}_1^2$ = 0.020, *P* = .888).

**Table 2:** Number of plants infected by Long-3’UTR, Short-3’UTR TMGMV isolates and/or STMV, per *N. glauca* population. Data are aggregated over collection of February 2018 and February 2020 for San José, La Datilera, rambla Morales, rambla Amoladeras, AL3115 and El Edar.

***Population***	***Not infected***	***Infected by***				***Total***
		***TMGMV***	***STMV***	***Long-3’UTR (+ STMV)***	***Short-3’UTR (+ STMV)***	
***San José***	*26*	*7*	*5*	*6 (5)*	*0 (0)*	*33*
***La Datilera***	*8*	*35*	*22*	*35 (35)*	*0 (0)*	*43*
***Rambla Morales***	*3*	*10*	*1*	*10 (1)*	*0 (0)*	*13*
***Rambla Amoladeras***	*53*	*10*	*7*	*4 (2)*	*6 (5)*	*63*
***AL3115***	*12*	*8*	*2*	*7 (1)*	*1 (1)*	*20*
***El Edar***	*7*	*28*	*18*	*0 (0)*	*28 (18)*	*35*
***Total***	*109*	*98*	*55*	*62 (44)*	*35 (24)*	*207*

The effect of STMV on the accumulation of TMGMV was analysed in the two *N. glauca* populations with the highest TMGMV and STMV prevalence in February 2020: La Datilera and El Edar (Table 3). A GLM considering population, STMV presence, and their interaction as fixed factors showed a significant effect of the population on TMGMV accumulation (*F*_1,32_ = 6.684, *P* = .014), but not of STMV presence (*F*_1,32_ = 0.367, *P* = .549) nor of the interaction term (*F*_1,32_ = 0.000, *P* = .998).

**Table 3:** Accumulation of TMGMV in the absence and presence of STMV in the populations of La Datilera and El Edar in the collections of February 2020. Data are shown as mean ng TMGMV RNA/μg total RNA and standard error over the different numbers of infected plants per collection.

***Population***	***STMV presence***	***TMGMV accumulation***
***La Datilera***	*Negative*	*79.383 ± 8.146*
	*Positive*	*90.728 ± 8.337*
***El Edar***	*Negative*	*53.054 ± 0.000* ^a^
	*Positive*	*64.326 ± 6.912*
***Total***	*Negative*	*70.607 ± 9.957*
	*Positive*	*76.751 ± 5.758*

^a^There was only one plant infected by TMGMV without STMV at El Edar population.

Thus, STMV presence does not have a significant effect on the accumulation of TMGMV, in the *N. glauca* populations of SE Spain.

The large variation of STMV prevalence in TMGMV-infected plants, both across populations (Table 2) and between years (18.20% in 2018 and 88.70% in 2020, Table S2) suggest different infection dynamics for TMGMV and STMV. This hypothesis was tested in the *N. glauca* population at Talud de Carboneras (Fig. 1), which was monitored from 1992 to 2003, allowing to study the dynamics of the *N. glauca* population and of TMGMV and STMV infection. The size of the *N. glauca* population changed during the studied period (Fig. 1A), increasing quickly during the time when the slope was colonized, and decreasing after 1994 as it was displaced by the vegetation of later successional communities. Table 4 and Table S3 show the number of plants infected by TMGMV of either a Long- or a Short-3’UTR and by STMV. The frequency of STMV in the TMGMV population was not associated with 3’UTR size (${\chi}_1^2$ = 0.710, *P* = .399), consistent with data from the six populations sampled in 2018 and 2020. Thus, the TMGMV Long-3′ or Short-3′ genotype is not further considered in this study. The prevalence of TMGMV (without and with STMV) increased over time, being 85.70% in 2003. STMV was detected later than TMGMV, and its incidence increased to a maximum of 57.70% over the *N. glauca* population (78.95% over the TMGMV-infected population) in January 1996, to decrease sharply afterwards. The TMGMV infection progress curve was adjusted to a logistic model (Fig. S2), which allowed to estimate an apparent infection rate *r_max_* = 0.0506 month^−1^.

**Figure 1:**
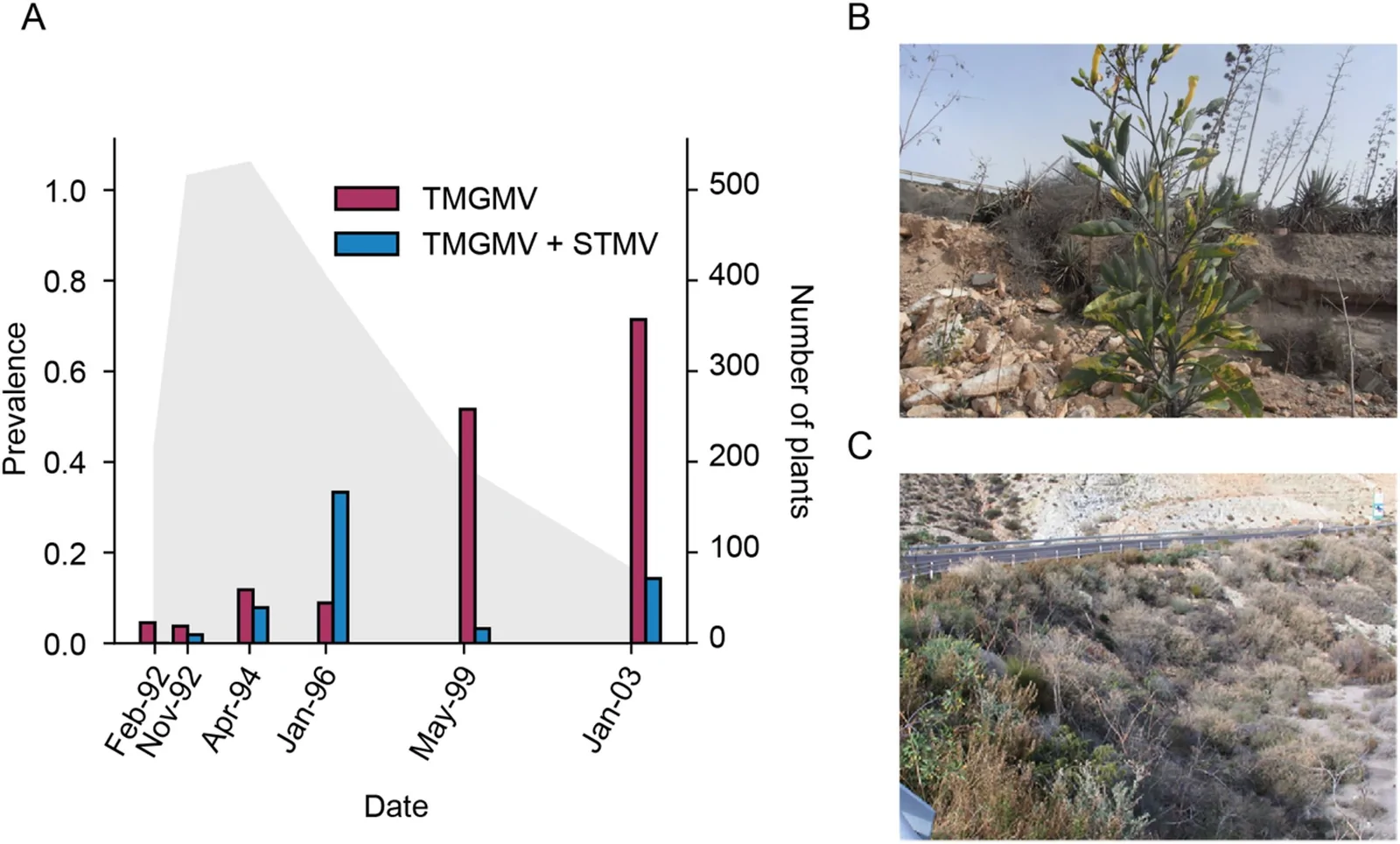
Variation of TMGMV and TMGMV + STMV prevalence in the *N. glauca* population of Talud de Carboneras. Panel A shows the prevalence of plants infected by TMGMV or by TMGMV + STMV, and the grey area represents the estimated number of plants of the population per timepoint. Panel B shows a symptomatic TMGMV-infected *N. glauca* plant. Panel C shows a view of the site in 2003, when the native xeric vegetation had mostly displaced *N. glauca*.

**Table 4:** Number of sampled *N. glauca* plants, and of plants infected by Long-3’UTR, Short-3’UTR isolates and/or STMV in Talud de Carboneras, per collection date.

***Date***	***Not infected***	***Infected by***				***Total***
		***TMGMV***	***Long-3’UTR***	***Short-3’UTR***	***STMV***	
***February 1992***	*21*	*1*	*1*	*0*	*0*	*22*
***November 1992***	*50*	*3*	*2*	*0*	*1*	*53*
***April 1994***	*41*	*10*	*6*	*3*	*4*	*51*
***January 1996***	*26*	*19*	*14*	*5*	*15*	*45*
***May 1999***	*14*	*17*	*12*	*5*	*1*	*31*
***January 2003***	*2*	*12*	*7*	*5*	*2*	*14*
***Total***	*154*	*62*	*42*	*18*	*23*	*216*

### Experimental estimation of the rate of contact transmission of TMGMV and STMV

The probability of STMV transmission per each event of TMGMV transmission was analysed using five TMGMV isolates from Talud de Carboneras supporting STMV. Sap from field-infected plants was inoculated into *N. tabacum* cv. Xanthi-nc leaves, where nll indicate a successful infection at a single infection site, that is, a single transmission event. The fraction of nll, which necessarily indicate TMGMV infection, that had STMV was evaluated by RT-PCR detection of STMV in RNA extracts from the nll or from *N. tabacum* cv. Samsun inoculated with a single nll. Depending on the isolate, STMV was detected in 0.30 to 0.65 of nll, with a mean across isolates of 0.420 ± 0.060. In the susceptible host, *N. tabacum* cv. Samsum, TMGMV was detected in all inoculated plants and, again, STMV was detected in a different fraction of infected plants depending on the isolate, ranging from 0.25 to 0.50, and averaging 0.380 ± 0.046. Thus, STMV assays in both a local-lesion and a susceptible host provide similar estimates of STMV transmission in about 40% of TMGMV transmission events.

To estimate the transmissibility of TMGMV and if it is influenced by STMV, each of three field isolates (Ng 96/5 of a Long-3’UTR, Ng 99/16 of a Short-3’UTR and Ng 94/5 of a Long-3’UTR with STMV) were inoculated in the first plant of rows of 10 *N. tabacum* cv. Samsun plants. The progress of infection along each row was monitored by the appearance of symptoms, by retroinoculation in *N. tabacum* cv. Xanthi-nc and the development of nll (Fig. 2 and Table S4) and, at the end of the experiment, by RT-PCR detection of TMGMV and STMV. All TMGMV infected plants in the Ng 94/5 rows were also infected by STMV. TMGMV infection showed a sudden cessation of transmission after the third plant in the case of isolate Ng 94/5, supporting STMV, while for the other two isolates transmission decreased over time until finally stopping at plants three to seven, depending on the replicated row. AUDPC values were calculated from data on the progress of infection as shown by nll induction after retroinoculation at the different dpi. An ANOVA with isolate as a fixed factor showed that AUDPC values were significantly higher for isolate Ng 96/5 and Ng 99/16 than for Ng 94/5 (*F*_1,5_ *> 6.394, P* < .044), with no differences between the isolates without STMV (*F*_1,5_ = 0.004, *P* = .953). Thus, data for isolates Ng 96/5 and Ng 99/16 were pooled for subsequent analyses (Fig. 2A).

**Figure 2:**
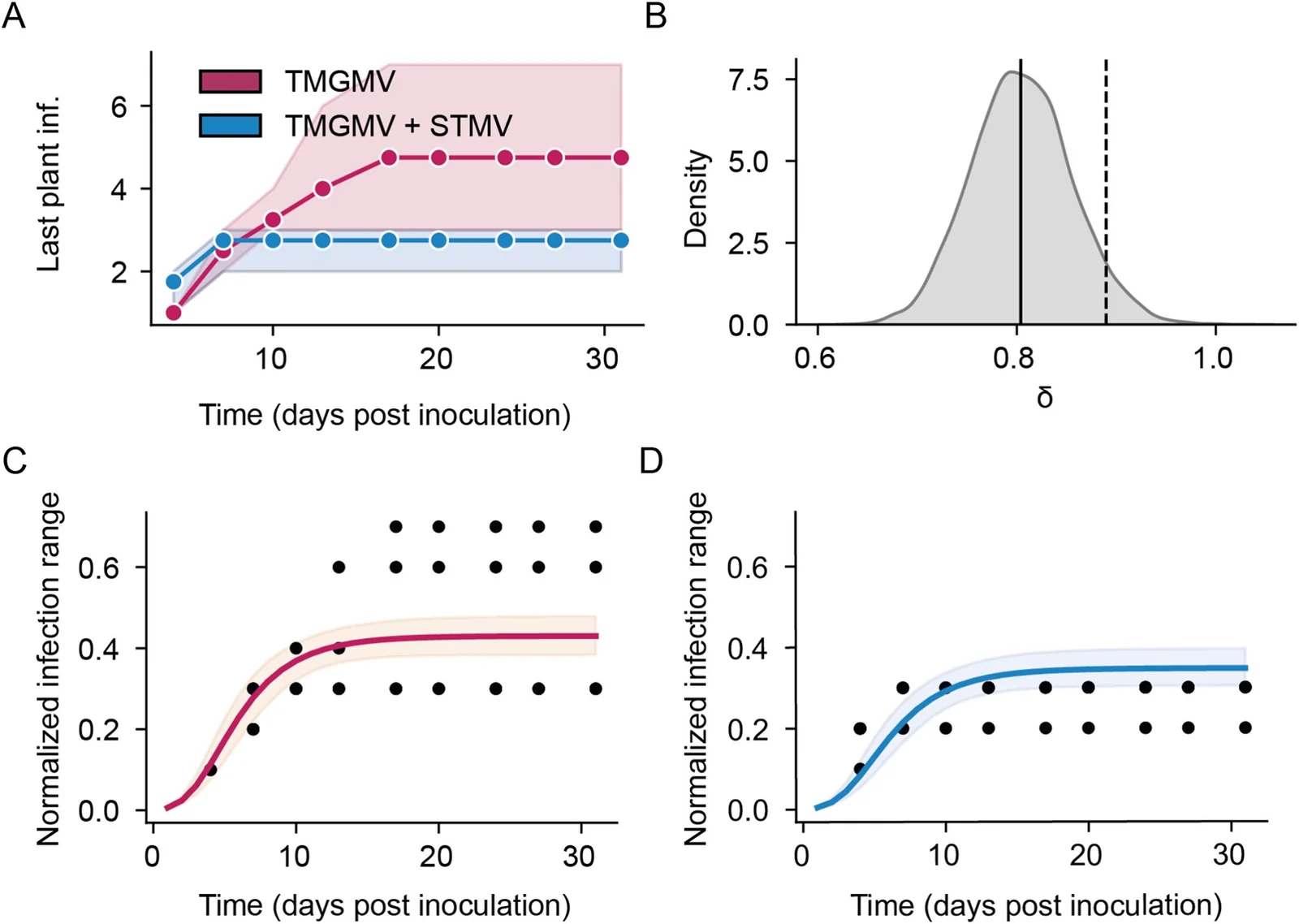
Experimental estimation of the contact transmission rate of TMGMV and STMV. A) Number of infected plants per row at different days-post-infection after initial inocculation with TMGMV or TMGMV + STMV. Shaded areas represent the minimum and maximum values observed in the four replicates. B) Markov chain Montecarlo (MCMC) distribution of *δ* (ratio between the transmission rates of TMGMV+STMV and TMGMV) after 1000 iterations in four chains. C) Model (Equation 2) predictions for TMGMV-inoculated data using the distributions of parameters obtained through MCMC. Shaded areas represent the 2.5% and 97.5% quantiles predicted. Black dots represent the corresponding experimental data D) model predictions in the TMGMV + STMV using the distributions of parameters obtained through MCMC. In panels C and D ‘Normalized infection range’ represents the number of infected plants, over 10 plants per replicated row, infected at any time post inoculation.

Quantification of TMGMV was performed at the inoculated plant (plant 1) of each row (Table S5). GLMs considering isolate as a fixed factor showed that the accumulation of Ng 96/5, of a Long-3’UTR was higher in both the inoculated and systemically infected leaves than that of Ng 99/16, of a Short-3’UTR (*F*_154_ > 23.717, *P* < .003). Ng 96/5 had a significantly higher accumulation than Ng 94/5, of a Long-3’UTR with STMV, in inoculated leaves (*F*_1,4_ = 14.711, *P* = .012), but not in upper leaves at seven dpi (*F*_1,5_ = 0.261, *P* = .628).

The data on the progress of infection along each row of plants (Fig. 2A) showed a clear decay of transmission over time and a lower transmission of the isolate supporting STMV than of the two isolates that do not support STMV. The ratio between *per capita* transmission rates from plants infected by TMGMV+SMTV and plants infected by TMGMV (*δ*) was estimated on two alternative models described by Equations 2 and 3, respectively. The Equation 2 model gave a median *δ =0.80* ([0.71–0.91] 95% CI), where the *δ* 97.5% quantile value (Fig. 2B) was below 1. The Equation 3 model provided a median of *δ =0.69* ([0.591, 0.807] 95% CI), whose 97.5% quantile was also below one. Therefore, we concluded that STMV produces a significant decrease of TMGMV transmission, with a median value between *δ =0.8* and *δ =0.7* depending on the choice of model. In the following sections, we use the median value of *δ =0.8*, but conclusions hold for *δ =0.7*.

### Dynamics of TMGMV and STMV infection in *N. glauca* populations

The field survey and experimental data in the sections before were used to implement simulations of the dynamics of TMGMV and STMV infection in *N. glauca* populations according to the mean field model described by Equation 1. We solve the states where the system reaches equilibrium $\dot{S}=\dot{I}=\dot{D}=0$, and Fig. 3A and 3B show for different values of the virulence *α,* the effect of variation in the values of ${\beta}_D$ and ${\beta}_I$ on the transitions between the phases where *S*, *I* and *D* plants are most prevalent. The separations between phases are non-linear, but for *S=I=D* (point *d* in Fig. 3A) the analytic solution (Equation 4) shows that when *γ =0.5* this point is reached when ${\beta}_D={\beta}_I$, and as the virulence decreases it moves closer to ${\beta}_I$= 0 (Fig. 3  A and B), making the region where *S* plants prevail smaller.


(4) \begin{align*} {\beta}_I&=6\mu -\frac{3\mu }{2\gamma}\nonumber\\{}{\beta}_D&=\frac{\beta_I}{4\gamma -1} \end{align*}

**Figure 3:**
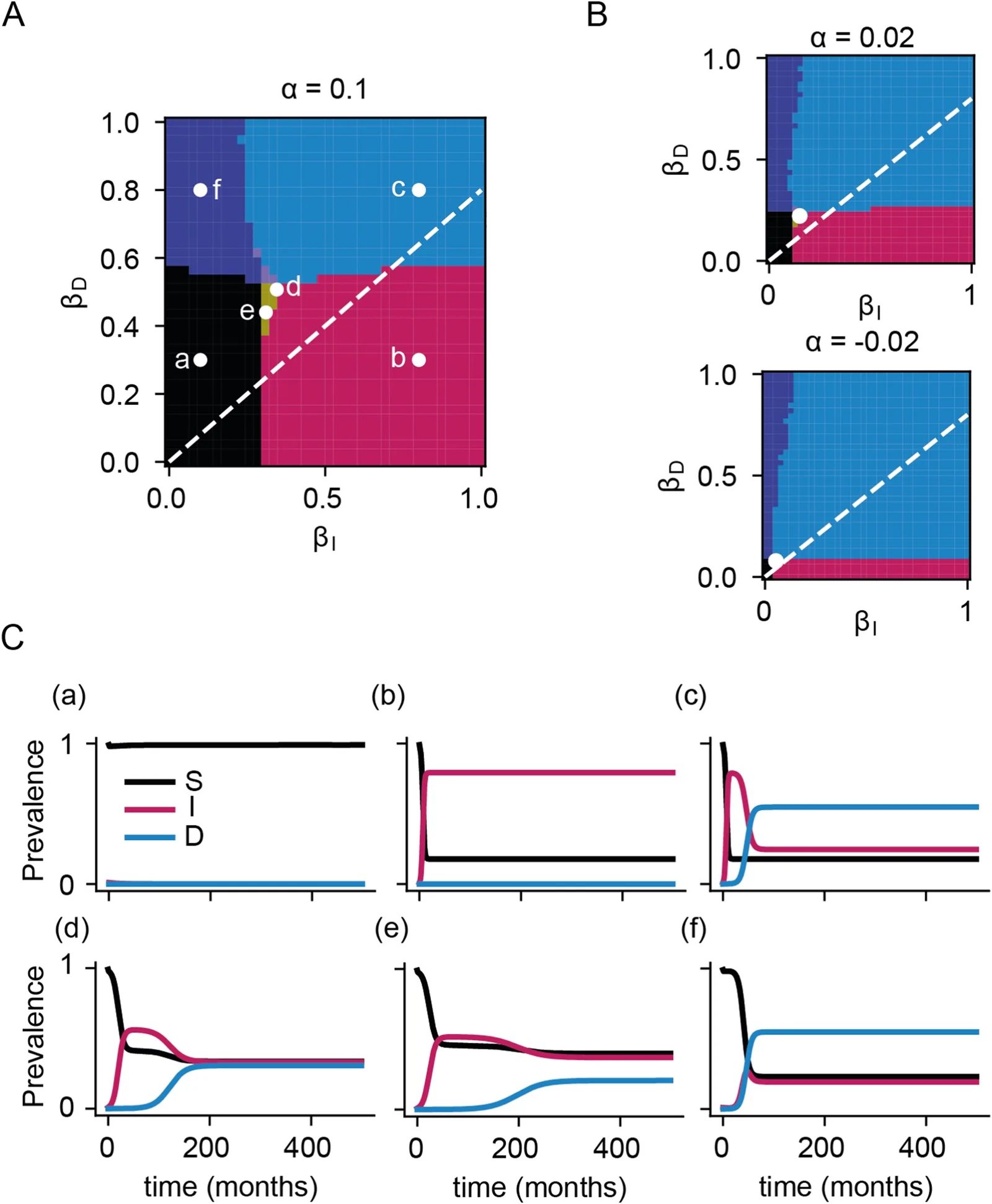
Predictions of infection dynamic states according to the Equation 1 model. A) Areas in which *S*, *I* or *D* plants are the most prevalent for different ${\beta}_I$ and ${\beta}_D$ values and *α =0.1*. Each area corresponds to a different dynamic before reaching the steady state. White points represent specific examples for which dynamics are shown in panel C. The white dashed line represents the value at which ${\beta}_D=\delta{\beta}_I$. B) Same as a, but at *α =-0.02* and *α =0.02*. C) Simulation dynamics for the highlighted dots in panel A. the dynamics of *S, I* and *D* plants are represented by black, red and blue lines, respectively.

Figure 3C shows the dynamics of *S*, *I* and *D* plants for the parameter values of the points highlighted in Fig. 3A. For point *a* (Fig. 3C(a)), susceptible plants prevail, as transmission is too low compared with mortality. Regions exemplified by points *b* and *c* show scenarios where *S* plants are replaced by *I* plants, or by *I* plants followed by *D* plants, respectively. In the small region exemplified by point *e, S* plants prevail in the long run after a period in which *I* plants are more frequent. Also note that at point *d*, the state *S=I=D*, is reached after a period when *I* plants prevail. Last, at point *f, D* plants prevail, but the steady state is reached with a different dynamic than at point *c*, as *I* plants never prevail.

The dotted white line in Fig. 3 A, B and C indicates the estimated 0.8 ratio between the values of ${\beta}_D$ and ${\beta}_I$. Under this condition, no matter the value of *α*, there are only three possibilities, *S* plants prevail, *I* plants prevail, or *D* plants prevail after an earlier prevalence of *I* plants. This is shown in Fig. 4A, with Fig. 4B showing the different dynamics associated to each point along the *α =0.1* line. It can be observed that prevalence phases are separated by straight lines, corresponding to *S=I* and *D=I* states.

**Figure 4:**
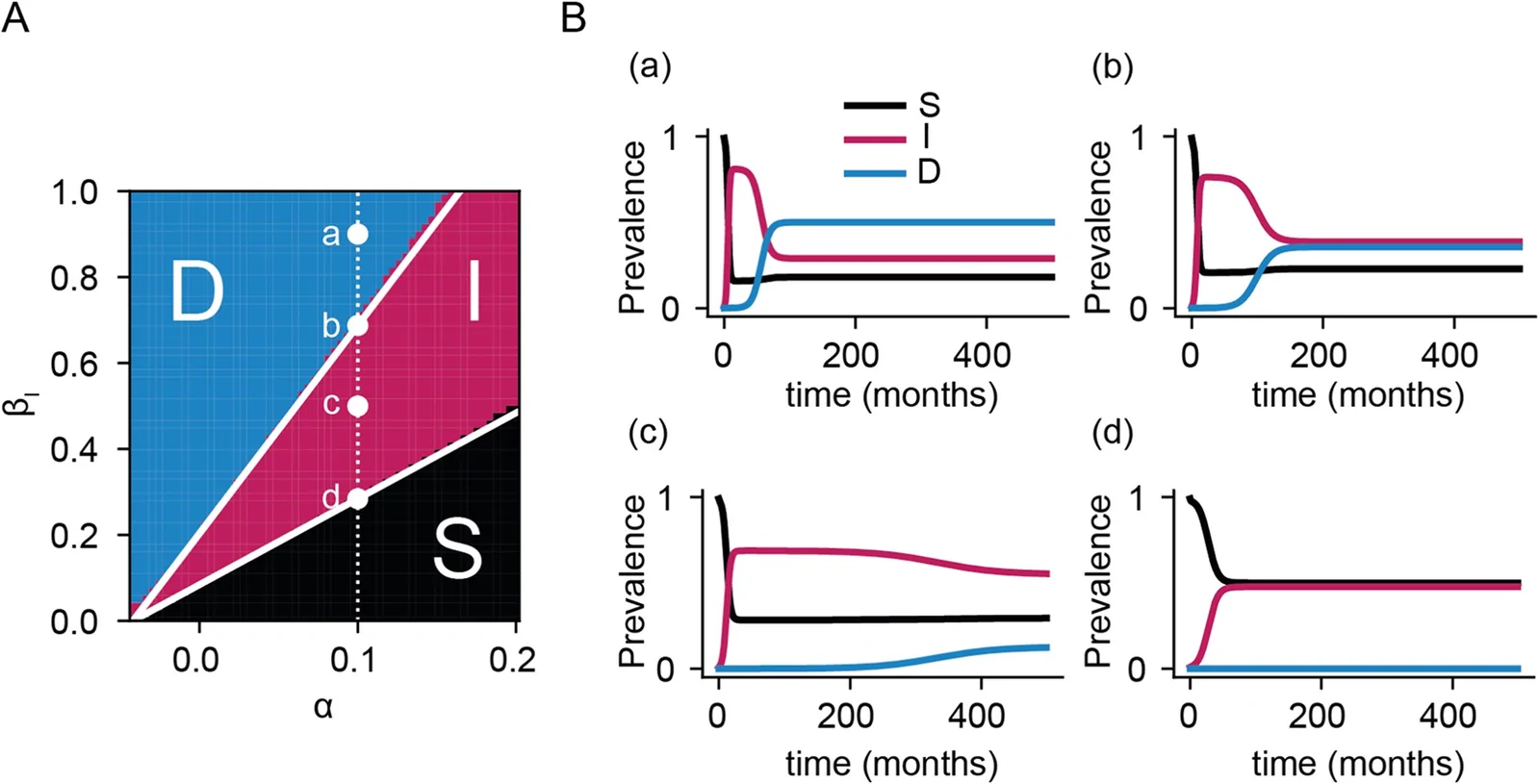
Predictions of infection dynamics states according to the Equation1 model for ${\beta}_D=0.8\ {\beta}_I$. A) Areas in which S, I or D plants are the most prevalent for different *α* and ${\beta}_I$ values. Each area corresponds to a different dynamic before reaching the steady state. White points represent specific examples for which dynamics are shown in panel B. B) Simulations dynamics for the highlighted dots in panel A. the dynamics of S, I and D plants are represented by black, red and blue lines, respectively.

The analytic solutions associated to *S=I* and *D=I* states show how the values of ${\beta}_I$ on these lines are proportional to *m+α* (see Supplementary Appendix A, Equations S1 and S2). In the case of the *S=I*, it can be shown that the equation is only defined when $\gamma <1/2\delta$ if *δ <1*. The experimentally determined values of *γ =0.42* and *δ =0.8* are consistent with these conditions. Fig. S3 illustrates how the value of *γ* influences the prevalence of *S, I* and *D*, and how the phase where *I* prevails disappears when the condition $\gamma <1/2\delta$ is not fulfilled.

In summary, under the observed ratio of ${\beta}_D$ to ${\beta}_I$ there is a linear relationship between *α* and ${\beta}_I$ that determines not only the stationary states but also the dynamics before reaching that sate, which are limited to three possibilities. The experimentally determined value of the *γ* parameter was below the limit (*1/2δ =0.625*) above which according to this model, *I* plants cannot prevail. Predictions on the conditions under which *D* plants prevail show that STMV cannot invade the TMGMV population except at low or negative values of virulence, a condition that can be met in the field. However, none of the possible dynamics predicted (Fig. 3 and Fig. 4) explain the transition between prevalence states observed in the field (Fig. 1A), where *D* plants initially prevail in the *N. glauca* population to be later displaced by *I* plants.

### Effects of variation of the *N. glauca* population on the infection dynamics of TMGMV and STMV

The model described by Equation 1 assumes a constant host population, which conditions model predictions. However, *N. glauca* populations are not constant and, if the habitat is not disturbed, decrease in time as the species is displaced by later successional vegetation, as shown in Fig. 1A. A decrease in population size will negatively affect virus transmission as the distance between infected and non-infected hosts is dependent on host density. To account for this effect, a time-dependent transmission factor ${\beta}_I(t)$ was introduced into the model. A sudden decrease in population density (i.e. of transmission rate) allows for *I* plants to take over in a host population in which *D* plants had become prevalent (Fig. 5A and B).

**Figure 5:**
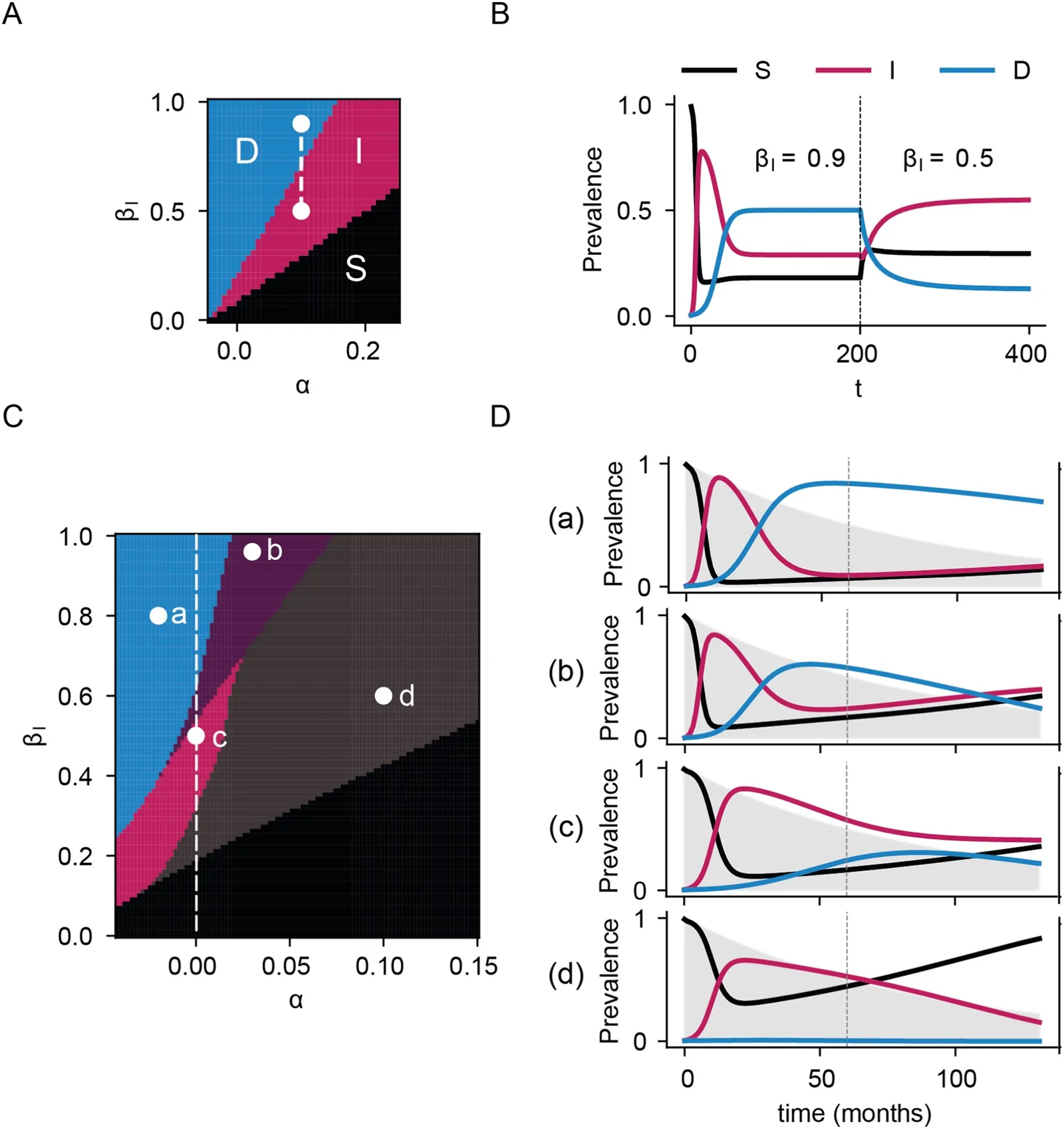
Predictions of infection dynamics states under varying host plant population densities that translate into varying transmission rates over time. A) Areas in which S, I or D plants are the most prevalent for different $\mathrm{\alpha}$ and ${\mathrm{\beta}}_{\mathrm{I}}$ values. Each area corresponds to a different dynamic during the first 132 months of simulation. White points represent specific examples for which dynamics are shown in panel B. B) Simulations dynamics under a transition between the highlighted dots in panel A at time t = 200. C) Areas of prevalence of S, I or D plants for different $\mathrm{\alpha}$ and initial ${\mathrm{\beta}}_{\mathrm{I}}$ values, under a 60 months half-life exponential decrease of transmission over time. Each area corresponds to a different dynamic during the first 132 months of simulation. White points represent specific examples for which dynamics are shown in panel D. D) Simulations dynamics for the highlighted dots in panel C. The shaded grey area represents the decay of transmission during the simulations the dynamics of S, I and D plants are represented by black, red and blue lines, respectively.

A smoother decrease in host plant density, such as in Fig. 1A, may be a more frequent scenario, which we modelled as an exponential decay of the transmission rate within the host plant population. This leads to a whole range of possible infection dynamics in a time frame of 11 years (Fig. 5C and D) according to the values of transmission rate and virulence, including the possibility of *D* plants becoming prevalent to be later displaced by *I* plants (Fig. 5D(b)). The results in Figure 5C and D show that a shrinking host population can lead to infection dynamics not predicted under a constant host population, with the most realistic ones occurring in regions of low to negative virulence.

In nature, a *N. glauca* population will initially increase in size while colonising a disturbed habitat (Fig. 1A). Incorporating a logistic growth model to model the increase of the transmission during the *N. glauca* population growing period does not predict new dynamics, but a delay in time of dynamics such as those in Fig. 4 (Fig. S4). Combining the growth and decrease of *N. glauca* populations in a single model does neither unveil new dynamics (Fig. S5).

## Discussion

Interest on the asymmetrical interactions between satellites and helper viruses has led to theoretical analyses of the conditions for satellite invasion and persistence, and of satellite impacts on the infection dynamics and evolution of helper viruses (e.g. Nee 2000, Zhang et al. 2000, Sofonea et al. 2015, Lucia-Sanz et al. 2022, Alizon 2023, Leeks et al. 2023, Lázaro et al. 2024). However, theory suffers from a lack of understanding of the ‘natural history’ of satellite helper virus associations, as research has rather focussed on their molecular biology (Nee 2000), and there is an almost complete lack of knowledge on the actual range of values of model parameters. In addition, predictions have rarely been tested with empirical data from nature (but see (Escriu et al. 2000, Betancourt et al. 2013).

The infection dynamics and genetic diversity of TMGMV in *N. glauca* populations has been much studied in Australia, Spain and California, the only region where STMV has been reported (Randles et al. 1981, Valverde and Dodds 1986, Rodríguez-Cerezo et al. 1991, Valverde 1991, Fraile et al. 1996, 1997, Bodaghi et al. 2004, de Andrés-Torán et al. 2025). The geographical range of STMV is now extended to Spain, where isolates were highly similar to the type isolate from California (Mirkov et al. 1989), and showed a low genetic diversity not structured according to the host plant population (Fig. S1), similarly to TMGMV (de Andrés-Torán et al. 2025). The average prevalence of STMV on TMGMV-infected plants was of 0.56, or about 0.39 in the whole *N. glauca* population, where TMGMV prevalence was ~ 0.70 (de Andrés-Torán et al. 2025), indicating a stable satellite-helper virus association. Interestingly, this figure is very similar to that reported in California (0.37 Valverde 1991), suggesting an upper threshold to STMV spread in the host plant population. In the studied area, prevalence of STMV varied largely in space (among *N. glauca* populations, Table 2) and time (Fig. 1 and Table S2) and did not parallel TMGMV prevalence, as for example in Talud de Carboneras, where the incidence of STMV increased to later decrease while that of TMGMV increased monotonically. These data indicate different infection dynamics of satellite and helper virus as well as the epidemic spread of STMV in the TMGMV population. Uncoupled infection dynamics and epidemic spread of the satellite in the helper virus population have also been reported for a largely different system, CMV and CMVsatRNA (Alonso-Prados et al. 1998), and could be a feature of satellite-helper virus associations. In *N. glauca* population STMV was not differentially associated with TMGMV isolates of a Long- or a Short-3’UTR, which differ in their infection dynamics (de Andrés-Torán et al. 2025). STMV did not affect TMGMV accumulation or symptom expression in field-infected plants, two features also observed in experiments (Valverde 1991). Thus, STMV has no, or a minor, impact on the accumulation and virulence of the helper virus, suggesting a commensal association (Lucia-Sanz et al. 2022). This is at odds with most analysed satellite viruses and satellite nucleic acids, which interfere with helper virus replication and modify its symptoms (Gnanasekaran and Chakraborty 2018). However, the prevalence of STMV was always lower than that of TMGMV, suggesting a less efficient transmission from plant to plant.

The above observed traits of the system were considered in a mean field model to explore the conditions of invasion and persistence of STMV. In this model, STMV can infected susceptible (*S*) plants together with TMGMV or plants already infected by TMGMV (*I* plants), i.e. can spread epidemically on the TMGMV population. Also, to account for the lower prevalence of STMV than TMGMV, the transmission rates from TMGMV and STMV+TMGMV-infected plants (*I* and *D* plants, respectively) might differ and the success of transmission of STMV per transmission event from *D* plants might be below one. Experiments showed indeed that STMV affected the transmission of TMGMV, transmission rates from STMV-infected plants being ~ 80% of those from plants infected only by TMGMV. This result did not depend on the virus accumulation in source plants, and is in apparent contradiction with the fact that STMV does not have a major effect on TMGMV accumulation (Valverde 1991, this work), as contact transmission of tobamoviruses depends on the virus titre in the source leaf (Sacristán et al. 2011). However, it can be explained by the fact that infection sites in a leaf are easily saturated (Siegel and Wildman 1956, Furumoto and Mickey 1967), and that STMV and TMGMV particles may compete for infection sites, with sites occupied only by STMV not producing an infection. The same argument explains the fact that STMV is not transmitted in all TMGMV transmission events (only in ~42%). Note that the values of transmission rate from STMV+TMGMV- relative to TMGMV-infected plants, and the success of STMV transmission per transmission event, also will depend on the relative accumulation of TMGMV and STMV in the source leaf, which we did not quantify.

Model simulations indicated that satellite and helper virus infection dynamics were highly dependent on the ratio between the transmission rates of *D* and *I* plants, on virulence, and on the realized rate of STMV transmission per TMGMV transmission event (Fig. 3, Fig. 4 and Fig. S3). Satellite invasion (*i.e.* higher prevalence of *D* plants) required transmission rates that were unrealistic according to the apparent infection rate estimated from the progress of infection at Talud de Carboneras, or/and low or negative values of virulence, which is compatible with the conditional mutualism shown for TMGMV and *N. glauca* in the analysed environment (de Andrés-Torán et al. 2025). A low virulence of the helper virus has been predicted to be required for the persistence of satellites that depend on a helper virus for transmission (Zhang et al. 2000). Our results extend this condition to a satellite that depends on the helper virus for replication. Under high transmission or/and low virulence, satellite invasion was subsequent to TMGMV invasion (Fig. 4), as was observed in the field (Fig. 1). This dynamic required values of the success of satellite transmission per transmission event (parameter γ) lower than 0.625, again in agreement with the experimental estimate of 0.42.

When the model was made to account for a decrease of host population size, it predicted dynamics in which the satellite would become extinct after invasion, as observed in the field. Interestingly, the host population density was a key factor in satellite invasion and persistence, which only occurred at host densities that translated into high transmission rates. The higher transmission rates from *I* plants will allow TMGMV to persist at lower *N. glauca* densities than STMV, again as observed. The patchy populations of *N. glauca* in SE Spain undergo large fluctuations of density, which increase when plants colonize newly disturbed habitats, and decrease gradually due to the efficient competition of the native vegetation. *N. glauca* populations can also decrease in size suddenly due to disturbances such as the occasional flood of the gullies that are the plant preferred habitats, which are later recolonised in a metapopulation-like dynamic (Rodríguez-Cerezo et al. 1991, MITECO 2020, de Andrés-Torán et al. 2025). The population dynamics of the host plant may favour the persistence of STMV, as it is predicted that coexistence of a virus and a dependent subviral element will be favoured under structured demes (Szathmáry 1992). However, observations of STMV persistence and host plant population dynamics do not agree with predictions that coexistence would be difficult under metapopulation dynamics (Nee 2000). Other factors not considered in our model analyses could also modulate STMV transmission dynamics, notably the presence of other helper viruses infecting the *N. glauca* populations. However, our extensive analyses of the systems during more than 40 years have failed to detect other tobamoviruses infecting *N. glauca* populations in SE Spain (unpublished).

A high transmission rate was also the main condition for the coexistence of CMV and CMVsatRNA, a satellite that competes with the helper virus for replication, severely depressing its within-host accumulation. However, a low virulence was not a condition for CMVsatRNA invasion and persistence (Betancourt et al. 2013, Escriu et al. 2003). The common need of high transmission rates for coexistence, which translate into the requirement of high plant density for the contact-transmitted STMV-TMGMV and high aphid density for the aphid-transmitted CMVsatRNA-CMV, derives from different within-host and between-host processes in each system, which need to be jointly considered when drawing predictions (Sofonea et al. 2015, Elena 2023). CMVsatRNA decreases CMV transmission as a result of a within-host process, its negative effect on CMV accumulation. STMV, on the contrary, decreases TMGMV as a result of a between-host process: the competition for a limited number of infection sites. We can hypothesize that interference with transmission will set an upper threshold to the within-host multiplication of all satellites. In the case of satellite nucleic acids, which do not encode a capsid and compete with the helper virus for replication and encapsidation, within-host multiplication interferes with their and the helper virus, transmission. In the case of satellite viruses, a high production of particles could block the transmission of the helper virus. Both CMVsatRNA and STMV, though, share the trait of a lower success of infection per transmission event than the helper virus (Escriu et al. 2000, Betancourt et al. 2011), this work). This could be a general feature of satellite—helper virus systems, as data on the only other satellite for which prevalence in the field has been estimated, satellite panicum mosaic virus (*Papanivirus panici*), indicates it is lower than for the helper panicum mosaic virus (*P. panici*), despite very efficient transmission in lawns by mowers (Cabrera and Scholthof 1999).

In conclusion, the comparison of the STMV-TMGMV and CMVsatRNA-CMV systems only provides partial support to the hypothesis that effects of satellites on helper virus replication will determine conditions for satellite—helper virus coexistence. Rather, we can hypothesize that a negative effect of satellites on the transmission of helper viruses is a general trait, regardless of the outcome of the within-host interaction, and, thus, no satellite—helper virus association would be truly neutral. More systems need be studied to test this hypothesis.

## Supplementary Material

Supplementary_materials_veag063

## Data Availability

All data generated and analysed in this study are included in this article and its supporting information files. Newly determined nucleotide sequences and other nucleotide sequences analysed are available in GenBank under accession numbers PZ470493 to PZ470501.
