# Supplementary material for: Conditions for invasion and persistence of a satellite that does not affect the replication and virulence of its helper virus
Source: Virus Evol. 2026 Sep 24;12(1):veag063. doi: 10.1093/ve/veag063 (PMC13652652; doi:10.1093/ve/veag063)
Supplement: Supplementary file 1 [file supplementary_materials_veag063.docx]

**Appendix A: Role of** $\boldsymbol{\gamma}$ **in the prevalences reached at the stationary state**

We obtained analytically the conditions under which the equilibrium prevalences $I=D$ and $S=I$ are observed at the stationary state, which delimit the prevalence phases in **Figure 4**. In the first case ($I=D$), the equation is:

$$\begin{aligned} \beta_{I}^{I=D}=\frac{2\left( \delta+1-\delta\gamma\right)}{\delta\gamma\left( \delta+1 \right)}\mu\#\left( S1 \right) \end{aligned}$$

where $\mu=m+ \alpha.$In the second case ($I=S$), the equation is:

$$\begin{aligned} \beta_{I}^{I=S}=2\mu\#\left( S2 \right) \end{aligned}$$

However, this solution only exists if $D=0$ at the stationary state. The solutions for $D>0$ are given by.

$$\beta_{I}^{D>0}=\frac{1}{\delta\gamma}\mu$$

Which can only be reached if

$$\begin{aligned} \gamma<\frac{1}{2\delta}\#\left( S3 \right) \end{aligned}$$

**Figure S3A** illustrates the relationship of $\beta_{I}^{D>0}$ and $\beta_{I}^{I=S}$, and the equilibrium values of *S*, *I* and *D* obtained for $\beta_{I}$ at different $\gamma$ for the observed $\delta=0.8$. In this plot, depending on the relative position of $\beta_{I}^{I=S}$ and $\beta_{I}^{D=0}$, **Equation S2** is defined ($\beta_{I}^{D>0}>\beta_{I}^{I=S}$) or undefined ($\beta_{I}^{D>0}<\beta_{I}^{I=S}$).

The $\gamma$ threshold value given by **Equation S3** is $\gamma=0.625$. **Figure** **S3B-D** illustrate how the prevalence phases change depending on the value of $\gamma$ and $\beta$ ($\alpha=0.1$). When $\gamma=0.42<1/2\delta$ (**Figure S3B**), there are three prevalence phases (*S, I,* and *D*). When $\gamma=0.625$ (**Figure S3C**), *I* does not prevail for any value of $\beta_{I}$, and is equal to *S* only in one point. When $\gamma>0.625$ (**Figure S3D**), *I* does not prevail for any value of $\beta_{I}$ and is never equal to *S*.

**Figure S1.** Maximum Likelihood phylogeny of the coat protein gene sequence from nine STMV isolates from *Nicotiana glauca* plants sampled in SE Spain from different populations and times, as indicated. Bootstrap percentage values > 40 are shown next to the corresponding node, and the highest likelihood (-602.17) is shown. STMV M25782.1 is a reference isolate from California, USA. Scale represents genetic distance as number of nucleotide substitutions per site. The tree is midpoint rooted.

**Figure S2**. **Progress of TMGMV infection in the *Nicotiana glauca* population Talud de Carboneras.** Data represent the prevalence f TMGMV plus TMGMV+STMV. Logistic regression fit to the The discontinuous line represents the fitted model. R^2^=0.572 *r_max_* = 0.0506 $\mathrm{month}^{-1}$.

**Figure S3**. **Predictions of infection dynamics states for different values of**  $\boldsymbol{\gamma}$. **A)** Areas in which *S, I* or *D* plants are the most prevalent for different $\alpha$ and $\beta_{I}$ values showing the boundaries of $\beta_{I}^{D>0}$ (dashed blue line) and $\beta_{I}^{I=S}$ (dashed orange line). Each area corresponds to a different steady state. The plot was obtained assuming $\gamma=0.42$ and $\delta=0.8$. **B-D)** Temporal variation of the prevalences of *D, S* and *I* plants at fixed $\alpha=0.1$ (white line on panel A) for **B)** $\gamma=0.42$ and $\delta=0.8$, **C)** $\gamma=0.625$ and $\delta=0.8$, and **D)** $\gamma=0.80$ and $\delta=0.8$.

**Figure S4:** **Predictions of infection dynamics states under increasing transmission rates over time.** **A)** Areas in which *S, I* or *D* plants are the most prevalent for different $\alpha$ and $\beta_{I}$ values under a logistic increase of the transmission over time with 60 months half-life and slope 0.083. Each area corresponds to a different dynamic during the first 132 months of simulation. White points represent specific examples for which dynamics are shown in panel **B**. **B)** Simulations dynamics for the highlighted dots in panel **A**. The shaded grey area represents the increase of transmission during the simulations The dynamics of S, I and D plants are represented by black, red and blue lines, respectively.

**Figure S5:** **Predictions of infection dynamics states under a combination of increase and decrease of transmission rates over time.** **A)** Areas in which *S, I* or *D* plants are the most prevalent for different $\alpha$ and $\beta_{I}$ values under changes of the transmission over time equal to the interpolation of the field data host population (**Figure 1**). Each area corresponds to a different dynamic during the first 132 months of simulation. White points represent specific examples for which dynamics are shown in panel **B**. **B)** Simulations dynamics for the highlighted dots in panel **A**. The shaded grey area represents the transmission values during the simulations. The dynamics of S, I and D plants are represented by black, red and blue lines, respectively.

**Table S1. Detection of STMV in TMGMV-infected *N. glauca* plants from six populations in SE Spain sampled in February 2018 and February 2020**. Data show for each individual plant, the sampling date, the population, the type of TMGMV isolate according to 3’UTR size, the STMV infection status and whether each plant showed symptoms (Mosaic) or not (None).

| ***Plant ID*** | ***Date*** | ***Population*** | ***3’UTR*** | ***STMV*** | ***Symptoms*** |
| --- | --- | --- | --- | --- | --- |
| *NgAL18.2* | *05/02/2018* | *Rambla Amoladeras* | *Long-3'UTR* | *Negative* | *None* |
| *NgAL18.3* | *05/02/2018* | *Rambla Amoladeras* | *Long-3'UTR* | *Negative* | *None* |
| *NgAL18.32* | *05/02/2018* | *Rambla Morales* | *Long-3'UTR* | *Negative* | *None* |
| *NgAL18.33* | *05/02/2018* | *Rambla Morales* | *Long-3'UTR* | *Negative* | *Mosaic* |
| *NgAL18.34* | *05/02/2018* | *Rambla Morales* | *Long-3'UTR* | *Negative* | *Mosaic* |
| *NgAL18.35* | *05/02/2018* | *Rambla Morales* | *Long-3'UTR* | *Negative* | *None* |
| *NgAL18.36* | *05/02/2018* | *Rambla Morales* | *Long-3'UTR* | *Negative* | *Mosaic* |
| *NgAL18.37* | *05/02/2018* | *Rambla Morales* | *Long-3'UTR* | *Negative* | *None* |
| *NgAL18.38* | *05/02/2018* | *Rambla Morales* | *Long-3'UTR* | *Positive* | *Mosaic* |
| *NgAL18.39* | *05/02/2018* | *Rambla Morales* | *Long-3'UTR* | *Negative* | *None* |
| *NgAL18.40* | *05/02/2018* | *Rambla Morales* | *Long-3'UTR* | *Negative* | *None* |
| *NgAL18.41* | *05/02/2018* | *Rambla Morales* | *Long-3'UTR* | *Negative* | *None* |
| *NgAL18.43* | *05/02/2018* | *La Datilera* | *Long-3'UTR* | *Negative* | *Mosaic* |
| *NgAL18.44* | *05/02/2018* | *La Datilera* | *Long-3'UTR* | *Negative* | *Mosaic* |
| *NgAL18.45* | *05/02/2018* | *La Datilera* | *Long-3'UTR* | *Negative* | *Mosaic* |
| *NgAL18.46* | *05/02/2018* | *La Datilera* | *Long-3'UTR* | *Negative* | *Mosaic* |
| *NgAL18.47* | *05/02/2018* | *La Datilera* | *Long-3'UTR* | *Negative* | *Mosaic* |
| *NgAL18.48* | *05/02/2018* | *La Datilera* | *Long-3'UTR* | *Negative* | *None* |
| *NgAL18.49* | *05/02/2018* | *La Datilera* | *Long-3'UTR* | *Negative* | *Mosaic* |
| *NgAL18.50* | *05/02/2018* | *La Datilera* | *Long-3'UTR* | *Negative* | *Mosaic* |
| *NgAL18.51* | *05/02/2018* | *La Datilera* | *Long-3'UTR* | *Positive* | *Mosaic* |
| *NgAL18.52* | *05/02/2018* | *La Datilera* | *Long-3'UTR* | *Negative* | *Mosaic* |
| *NgAL18.53* | *05/02/2018* | *La Datilera* | *Long-3'UTR* | *Positive* | *Mosaic* |
| *NgAL18.54* | *05/02/2018* | *La Datilera* | *Long-3'UTR* | *Negative* | *Mosaic* |
| *NgAL18.56* | *05/02/2018* | *La Datilera* | *Long-3'UTR* | *Positive* | *Mosaic* |
| *NgAL18.58* | *05/02/2018* | *La Datilera* | *Long-3'UTR* | *Positive* | *Mosaic* |
| *NgAL18.59* | *05/02/2018* | *La Datilera* | *Long-3'UTR* | *Negative* | *Mosaic* |
| *NgAL18.62* | *05/02/2018* | *La Datilera* | *Long-3'UTR* | *Positive* | *None* |
| *NgAL18.63* | *05/02/2018* | *La Datilera* | *Long-3'UTR* | *Positive* | *Mosaic* |
| *NgAL18.73* | *05/02/2018* | *San José* | *Long-3'UTR* | *Negative* | *None* |
| *NgAL18.75* | *05/02/2018* | *San José* | *Long-3'UTR* | *Positive* | *None* |
| *NgAL18.77* | *05/02/2018* | *AL3115* | *Long-3'UTR* | *Negative* | *None* |
| *NgAL18.78* | *05/02/2018* | *AL3115* | *Long-3'UTR* | *Negative* | *None* |
| *NgAL18.79* | *05/02/2018* | *AL3115* | *Long-3'UTR* | *Negative* | *None* |
| *NgAL18.81* | *05/02/2018* | *AL3115* | *Long-3'UTR* | *Negative* | *None* |
| *NgAL18.88* | *05/02/2018* | *El Edar* | *Short-3'UTR* | *Negative* | *Mosaic* |
| *NgAL18.90* | *05/02/2018* | *El Edar* | *Short-3'UTR* | *Negative* | *None* |
| *NgAL18.91* | *05/02/2018* | *El Edar* | *Short-3'UTR* | *Negative* | *Mosaic* |
| *NgAL18.92* | *05/02/2018* | *El Edar* | *Short-3'UTR* | *Negative* | *None* |
| *NgAL18.93* | *05/02/2018* | *El Edar* | *Short-3'UTR* | *Negative* | *None* |
| *NgAL18.95* | *05/02/2018* | *El Edar* | *Short-3'UTR* | *Negative* | *None* |
| *NgAL18.96* | *05/02/2018* | *El Edar* | *Short-3'UTR* | *Negative* | *Mosaic* |
| *NgAL18.97* | *05/02/2018* | *El Edar* | *Short-3'UTR* | *Negative* | *None* |
| *NgAL18.98* | *05/02/2018* | *El Edar* | *Short-3'UTR* | *Negative* | *Mosaic* |
| *NgAL18.632* | *18/02/2020* | *San José* | *Long-3'UTR* | *Positive* | *None* |
| *NgAL18.633* | *18/02/2020* | *San José* | *Long-3'UTR* | *Positive* | *None* |
| *NgAL18.635* | *18/02/2020* | *San José* | *Long-3'UTR* | *Positive* | *Mosaic* |
| *NgAL18.639* | *18/02/2020* | *San José* | *Long-3'UTR* | *Positive* | *Mosaic* |
| *NgAL18.647* | *18/02/2020* | *La Datilera* | *Long-3'UTR* | *Negative* | *None* |
| *NgAL18.648* | *18/02/2020* | *La Datilera* | *Long-3'UTR* | *Positive* | *None* |
| *NgAL18.649* | *18/02/2020* | *La Datilera* | *Long-3'UTR* | *Positive* | *Mosaic* |
| *NgAL18.650* | *18/02/2020* | *La Datilera* | *Long-3'UTR* | *Positive* | *Mosaic* |
| *NgAL18.651* | *18/02/2020* | *La Datilera* | *Long-3'UTR* | *Positive* | *Mosaic* |
| *NgAL18.652* | *18/02/2020* | *La Datilera* | *Long-3'UTR* | *Negative* | *Mosaic* |
| *NgAL18.653* | *18/02/2020* | *La Datilera* | *Long-3'UTR* | *Positive* | *Mosaic* |
| *NgAL18.654* | *18/02/2020* | *La Datilera* | *Long-3'UTR* | *Positive* | *Mosaic* |
| *NgAL18.656* | *18/02/2020* | *La Datilera* | *Long-3'UTR* | *Positive* | *Mosaic* |
| *NgAL18.657* | *18/02/2020* | *La Datilera* | *Long-3'UTR* | *Positive* | *Mosaic* |
| *NgAL18.658* | *18/02/2020* | *La Datilera* | *Long-3'UTR* | *Positive* | *Mosaic* |
| *NgAL18.659* | *18/02/2020* | *La Datilera* | *Long-3'UTR* | *Positive* | *None* |
| *NgAL18.660* | *18/02/2020* | *La Datilera* | *Long-3'UTR* | *Positive* | *Mosaic* |
| *NgAL18.662* | *18/02/2020* | *La Datilera* | *Long-3'UTR* | *Positive* | *Mosaic* |
| *NgAL18.663* | *18/02/2020* | *La Datilera* | *Long-3'UTR* | *Positive* | *Mosaic* |
| *NgAL18.664* | *18/02/2020* | *La Datilera* | *Long-3'UTR* | *Positive* | *None* |
| *NgAL18.666* | *18/02/2020* | *La Datilera* | *Long-3'UTR* | *Positive* | *Mosaic* |
| *NgAL18.667* | *18/02/2020* | *La Datilera* | *Long-3'UTR* | *Positive* | *Mosaic* |
| *NgAL18.671* | *18/02/2020* | *Rambla Amoladeras* | *Long-3'UTR* | *Positive* | *None* |
| *NgAL18.674* | *18/02/2020* | *Rambla Amoladeras* | *Long-3'UTR* | *Positive* | *None* |
| *NgAL18.693* | *18/02/2020* | *Rambla Amoladeras* | *Short-3'UTR* | *Negative* | *Mosaic* |
| *NgAL18.694* | *18/02/2020* | *Rambla Amoladeras* | *Short-3'UTR* | *Positive* | *Mosaic* |
| *NgAL18.695* | *18/02/2020* | *Rambla Amoladeras* | *Short-3'UTR* | *Positive* | *Mosaic* |
| *NgAL18.698* | *18/02/2020* | *Rambla Amoladeras* | *Short-3'UTR* | *Positive* | *Mosaic* |
| *NgAL18.699* | *18/02/2020* | *Rambla Amoladeras* | *Short-3'UTR* | *Positive* | *Mosaic* |
| *NgAL18.701* | *18/02/2020* | *Rambla Amoladeras* | *Short-3'UTR* | *Positive* | *Mosaic* |
| *NgAL18.704* | *18/02/2020* | *AL3115* | *Long-3'UTR* | *Negative* | *Mosaic* |
| *NgAL18.705* | *18/02/2020* | *AL3115* | *Long-3'UTR* | *Positive* | *Mosaic* |
| *NgAL18.706* | *18/02/2020* | *AL3115* | *Long-3'UTR* | *Negative* | *None* |
| *NgAL18.713* | *18/02/2020* | *AL3115* | *Short-3'UTR* | *Positive* | *Mosaic* |
| *NgAL18.714* | *18/02/2020* | *El Edar* | *Short-3'UTR* | *Positive* | *None* |
| *NgAL18.715* | *18/02/2020* | *El Edar* | *Short-3'UTR* | *Positive* | *None* |
| *NgAL18.716* | *18/02/2020* | *El Edar* | *Short-3'UTR* | *Positive* | *None* |
| *NgAL18.717* | *18/02/2020* | *El Edar* | *Short-3'UTR* | *Positive* | *Mosaic* |
| *NgAL18.718* | *18/02/2020* | *El Edar* | *Short-3'UTR* | *Positive* | *Mosaic* |
| *NgAL18.719* | *18/02/2020* | *El Edar* | *Short-3'UTR* | *Positive* | *Mosaic* |
| *NgAL18.720* | *18/02/2020* | *El Edar* | *Short-3'UTR* | *Positive* | *None* |
| *NgAL18.721* | *18/02/2020* | *El Edar* | *Short-3'UTR* | *Positive* | *None* |
| *NgAL18.724* | *18/02/2020* | *El Edar* | *Short-3'UTR* | *Positive* | *Mosaic* |
| *NgAL18.725* | *18/02/2020* | *El Edar* | *Short-3'UTR* | *Positive* | *Mosaic* |
| *NgAL18.729* | *18/02/2020* | *El Edar* | *Short-3'UTR* | *Positive* | *Mosaic* |
| *NgAL18.730* | *18/02/2020* | *El Edar* | *Short-3'UTR* | *Positive* | *Mosaic* |
| *NgAL18.731* | *18/02/2020* | *El Edar* | *Short-3'UTR* | *Positive* | *Mosaic* |
| *NgAL18.732* | *18/02/2020* | *El Edar* | *Short-3'UTR* | *Positive* | *None* |
| *NgAL18.733* | *18/02/2020* | *El Edar* | *Short-3'UTR* | *Positive* | *Mosaic* |
| *NgAL18.734* | *18/02/2020* | *El Edar* | *Short-3'UTR* | *Positive* | *Mosaic* |
| *NgAL18.735* | *18/02/2020* | *El Edar* | *Short-3'UTR* | *Positive* | *Mosaic* |
| *NgAL18.736* | *18/02/2020* | *El Edar* | *Short-3'UTR* | *Positive* | *Mosaic* |
| *NgAL18.737* | *18/02/2020* | *El Edar* | *Short-3'UTR* | *Negative* | *Mosaic* |

**Table S2. Number of plants infected by TMGMV and/or STMV, per *N. glauca* population**. Data are given separately for collections of February 2018 and February 2020 for San José, La Datilera, Rambla Morales, Rambla Amoladeras, AL3115 and El Edar.

| ***Population*** | ***2018*** | |  | ***2020*** | |  |
| --- | --- | --- | --- | --- | --- | --- |
|  | ***TMGMV*** | ***STMV*** | ***TMGMV*** | | ***STMV*** | |
| ***San José*** | 2 | 1 | 4 | | 4 | |
| ***La Datilera*** | 17 | 6 | 18 | | 16 | |
| ***Rambla Morales*** | 10 | 1 | 0 | | 0 | |
| ***Rambla Amoladeras*** | 2 | 0 | 8 | | 7 | |
| ***AL3115*** | 4 | 0 | 4 | | 2 | |
| ***El Edar*** | 9 | 0 | 19 | | 18 | |
| ***Total*** | **44** | **8** | **53** | | **47** | |

**Table S3. *N. glauca* plants from Talud de Carboneras in which TMGMV and STMV infection was evaluated**. For reach invidvidual plant, data show for each individual plant, the sampling date, the TMGMV infection status, the type of TMGMV isolate based on 3’UTR size and the STMV infection status.

| ***Plant ID*** | ***Date*** | ***TMGMV*** | ***3’UTR*** | ***STMV*** |
| --- | --- | --- | --- | --- |
| *Ng 92/4* | *02/1992* | *Negative* | *-* | *-* |
| *Ng 92/5* | *02/1992* | *Negative* | *-* | *-* |
| *Ng 92/6* | *02/1992* | *Negative* | *-* | *-* |
| *Ng 92/7* | *02/1992* | *Negative* | *-* | *-* |
| *Ng 92/8* | *02/1992* | *Negative* | *-* | *-* |
| *Ng 92/9* | *02/1992* | *Negative* | *-* | *-* |
| *Ng 92/10* | *02/1992* | *Negative* | *-* | *-* |
| *Ng 92/11* | *02/1992* | *Negative* | *-* | *-* |
| *Ng 92/12* | *02/1992* | *Negative* | *-* | *-* |
| *Ng 92/13* | *02/1992* | *Negative* | *-* | *-* |
| *Ng 92/14* | *02/1992* | *Negative* | *-* | *-* |
| *Ng 92/15* | *02/1992* | *Negative* | *-* | *-* |
| *Ng 92/16* | *02/1992* | *Negative* | *-* | *-* |
| *Ng 92/17* | *02/1992* | *Negative* | *-* | *-* |
| *Ng 92/18* | *02/1992* | *Negative* | *-* | *-* |
| *Ng 92/19* | *02/1992* | *Positive* | *Long-3'UTR* | *Negative* |
| *Ng 92/20* | *02/1992* | *Negative* | *-* | *-* |
| *Ng 92/21* | *02/1992* | *Negative* | *-* | *-* |
| *Ng 92/22* | *02/1992* | *Negative* | *-* | *-* |
| *Ng 92/23* | *02/1992* | *Negative* | *-* | *-* |
| *Ng 92/24* | *02/1992* | *Negative* | *-* | *-* |
| *Ng 92/25* | *11/1992* | *Negative* | *-* | *-* |
| *Ng 92/26* | *11/1992* | *Negative* | *-* | *-* |
| *Ng 92/27* | *11/1992* | *Negative* | *-* | *-* |
| *Ng 92/28* | *11/1992* | *Negative* | *-* | *-* |
| *Ng 92/29* | *11/1992* | *Negative* | *-* | *-* |
| *Ng 92/30* | *11/1992* | *Negative* | *-* | *-* |
| *Ng 92/31* | *11/1992* | *Negative* | *-* | *-* |
| *Ng 92/32* | *11/1992* | *Negative* | *-* | *-* |
| *Ng 92/33* | *11/1992* | *Negative* | *-* | *-* |
| *Ng 92/34* | *11/1992* | *Negative* | *-* | *-* |
| *Ng 92/35* | *11/1992* | *Negative* | *-* | *-* |
| *Ng 92/36* | *11/1992* | *Negative* | *-* | *-* |
| *Ng 92/37* | *11/1992* | *Negative* | *-* | *-* |
| *Ng 92/38* | *11/1992* | *Negative* | *-* | *-* |
| *Ng 92/39* | *11/1992* | *Positive* | *Mixed* | *Positive* |
| *Ng 92/40* | *11/1992* | *Negative* | *-* | *-* |
| *Ng 92/41* | *11/1992* | *Negative* | *-* | *-* |
| *Ng 92/42* | *11/1992* | *Negative* | *-* | *-* |
| *Ng 92/43* | *11/1992* | *Negative* | *-* | *-* |
| *Ng 92/44* | *11/1992* | *Negative* | *-* | *-* |
| *Ng 92/45* | *11/1992* | *Negative* | *-* | *-* |
| *Ng 92/46* | *11/1992* | *Negative* | *-* | *-* |
| *Ng 92/47* | *11/1992* | *Negative* | *-* | *-* |
| *Ng 92/48* | *11/1992* | *Negative* | *-* | *-* |
| *Ng 92/49* | *11/1992* | *Negative* | *-* | *-* |
| *Ng 92/50* | *11/1992* | *Negative* | *-* | *-* |
| *Ng 92/51* | *11/1992* | *Negative* | *-* | *-* |
| *Ng 92/52* | *11/1992* | *Negative* | *-* | *-* |
| *Ng 92/53* | *11/1992* | *Negative* | *-* | *-* |
| *Ng 92/54* | *11/1992* | *Negative* | *-* | *-* |
| *Ng 92/55* | *11/1992* | *Positive* | *Long-3'UTR* | *Negative* |
| *Ng 92/56* | *11/1992* | *Negative* | *-* | *-* |
| *Ng 92/57* | *11/1992* | *Negative* | *-* | *-* |
| *Ng 92/58* | *11/1992* | *Negative* | *-* | *-* |
| *Ng 92/59* | *11/1992* | *Negative* | *-* | *-* |
| *Ng 92/60* | *11/1992* | *Negative* | *-* | *-* |
| *Ng 92/61* | *11/1992* | *Negative* | *-* | *-* |
| *Ng 92/62* | *11/1992* | *Negative* | *-* | *-* |
| *Ng 92/63* | *11/1992* | *Negative* | *-* | *-* |
| *Ng 92/64* | *11/1992* | *Negative* | *-* | *-* |
| *Ng 92/65* | *11/1992* | *Negative* | *-* | *-* |
| *Ng 92/66* | *11/1992* | *Negative* | *-* | *-* |
| *Ng 92/67* | *11/1992* | *Negative* | *-* | *-* |
| *Ng 92/68* | *11/1992* | *Negative* | *-* | *-* |
| *Ng 92/69* | *11/1992* | *Negative* | *-* | *-* |
| *Ng 92/70* | *11/1992* | *Negative* | *-* | *-* |
| *Ng 92/71* | *11/1992* | *Negative* | *-* | *-* |
| *Ng 92/72* | *11/1992* | *Negative* | *-* | *-* |
| *Ng 92/73* | *11/1992* | *Positive* | *Long-3'UTR* | *Negative* |
| *Ng 92/74* | *11/1992* | *Negative* | *-* | *-* |
| *Ng 92/75* | *11/1992* | *Negative* | *-* | *-* |
| *Ng 92/76* | *11/1992* | *Negative* | *-* | *-* |
| *Ng 92/77* | *11/1992* | *Negative* | *-* | *-* |
| *Ng 94/1* | *04/1994* | *Positive* | *Short-3'UTR* | *Positive* |
| *Ng 94/2* | *04/1994* | *Negative* | *-* | *-* |
| *Ng 94/3* | *04/1994* | *Positive* | *Mixed* | *Negative* |
| *Ng 94/4* | *04/1994* | *Positive* | *Long-3'UTR* | *Negative* |
| *Ng 94/5* | *04/1994* | *Positive* | *Long-3'UTR* | *Positive* |
| *Ng 94/6* | *04/1994* | *Negative* | *-* | *-* |
| *Ng 94/7* | *04/1994* | *Positive* | *Long-3'UTR* | *Negative* |
| *Ng 94/8* | *04/1994* | *Positive* | *Long-3'UTR* | *Negative* |
| *Ng 94/9* | *04/1994* | *Positive* | *Long-3'UTR* | *Negative* |
| *Ng 94/10* | *04/1994* | *Positive* | *Long-3'UTR* | *Positive* |
| *Ng 94/11* | *04/1994* | *Positive* | *Short-3'UTR* | *Positive* |
| *Ng 94/12* | *04/1994* | *Positive* | *Short-3'UTR* | *Negative* |
| *Ng 96/1* | *01/1996* | *Negative* | *-* | *-* |
| *Ng 96/2* | *01/1996* | *Negative* | *-* | *-* |
| *Ng 96/3* | *01/1996* | *Negative* | *-* | *-* |
| *Ng 96/4* | *01/1996* | *Negative* | *-* | *-* |
| *Ng 96/5* | *01/1996* | *Positive* | *Long-3'UTR* | *Positive* |
| *Ng 96/6* | *01/1996* | *Negative* | *-* | *-* |
| *Ng 96/7* | *01/1996* | *Negative* | *-* | *-* |
| *Ng 96/8* | *01/1996* | *Negative* | *-* | *-* |
| *Ng 96/9* | *01/1996* | *Positive* | *Long-3'UTR* | *Positive* |
| *Ng 96/10* | *01/1996* | *Negative* | *-* | *-* |
| *Ng 96/11* | *01/1996* | *Positive* | *Long-3'UTR* | *Positive* |
| *Ng 96/12* | *01/1996* | *Positive* | *Long-3'UTR* | *Positive* |
| *Ng 96/13* | *01/1996* | *Positive* | *Long-3'UTR* | *Negative* |
| *Ng 96/14* | *01/1996* | *Positive* | *Long-3'UTR* | *Positive* |
| *Ng 96/15* | *01/1996* | *Positive* | *Short-3'UTR* | *Positive* |
| *Ng 96/16* | *01/1996* | *Positive* | *Short-3'UTR* | *Positive* |
| *Ng 96/17* | *01/1996* | *Positive* | *Short-3'UTR* | *Negative* |
| *Ng 96/18* | *01/1996* | *Positive* | *Long-3'UTR* | *Positive* |
| *Ng 96/19* | *01/1996* | *Positive* | *Short-3'UTR* | *Negative* |
| *Ng 96/20* | *01/1996* | *Positive* | *Long-3'UTR* | *Positive* |
| *Ng 96/21* | *01/1996* | *Positive* | *Long-3'UTR* | *Positive* |
| *Ng 96/22* | *01/1996* | *Positive* | *Long-3'UTR* | *Positive* |
| *Ng 96/23* | *01/1996* | *Positive* | *Short-3'UTR* | *Positive* |
| *Ng 96/24* | *01/1996* | *Positive* | *Long-3'UTR* | *Positive* |
| *Ng 96/25* | *01/1996* | *Positive* | *Long-3'UTR* | *Negative* |
| *Ng 96/26* | *01/1996* | *Positive* | *Long-3'UTR* | *Positive* |
| *Ng 96/27* | *01/1996* | *Positive* | *Long-3'UTR* | *Positive* |
| *Ng 96/28* | *01/1996* | *Negative* | *-* | *-* |
| *Ng 99/1* | *05/1999* | *Positive* | *Long-3'UTR* | *Negative* |
| *Ng 99/2* | *05/1999* | *Positive* | *Long-3'UTR* | *Negative* |
| *Ng 99/3* | *05/1999* | *Positive* | *Short-3'UTR* | *Negative* |
| *Ng 99/4* | *05/1999* | *Negative* | *-* | *-* |
| *Ng 99/5* | *05/1999* | *Negative* | *-* | *-* |
| *Ng 99/6* | *05/1999* | *Positive* | *Long-3'UTR* | *Negative* |
| *Ng 99/7* | *05/1999* | *Positive* | *Long-3'UTR* | *Negative* |
| *Ng 99/8* | *05/1999* | *Negative* | *-* | *-* |
| *Ng 99/9* | *05/1999* | *Positive* | *Short-3'UTR* | *Negative* |
| *Ng 99/10* | *05/1999* | *Positive* | *Short-3'UTR* | *Negative* |
| *Ng 99/11* | *05/1999* | *Positive* | *Long-3'UTR* | *Negative* |
| *Ng 99/12* | *05/1999* | *Negative* | *-* | *-* |
| *Ng 99/13* | *05/1999* | *Positive* | *Long-3'UTR* | *Negative* |
| *Ng 99/14* | *05/1999* | *Positive* | *Long-3'UTR* | *Negative* |
| *Ng 99/15* | *05/1999* | *Positive* | *Long-3'UTR* | *Negative* |
| *Ng 99/16* | *05/1999* | *Positive* | *Short-3'UTR* | *Negative* |
| *Ng 99/17* | *05/1999* | *Positive* | *Long-3'UTR* | *Negative* |
| *Ng 99/18* | *05/1999* | *Positive* | *Long-3'UTR* | *Positive* |
| *Ng 99/19* | *05/1999* | *Positive* | *Long-3'UTR* | *Negative* |
| *Ng 99/20* | *05/1999* | *Positive* | *Long-3'UTR* | *Negative* |
| *Ng 99/21* | *05/1999* | *Positive* | *Short-3'UTR* | *Negative* |
| *Ng 03/1* | *01/2003* | *Positive* | *Short-3'UTR* | *Negative* |
| *Ng 03/2* | *01/2003* | *Positive* | *Short-3'UTR* | *Negative* |
| *Ng 03/3* | *01/2003* | *Positive* | *Long-3'UTR* | *Negative* |
| *Ng 03/4* | *01/2003* | *Positive* | *Long-3'UTR* | *Positive* |
| *Ng 03/5* | *01/2003* | *Positive* | *Long-3'UTR* | *Positive* |
| *Ng 03/6* | *01/2003* | *Positive* | *Long-3'UTR* | *Negative* |
| *Ng 03/7* | *01/2003* | *Positive* | *Long-3'UTR* | *Negative* |
| *Ng 03/8* | *01/2003* | *Positive* | *Long-3'UTR* | *Negative* |
| *Ng 03/9* | *01/2003* | *Positive* | *Short-3'UTR* | *Negative* |
| *Ng 03/10* | *01/2003* | *Positive* | *Short-3'UTR* | *Negative* |
| *Ng 03/11* | *01/2003* | *-* | *-* | *-* |
| *Ng 03/12* | *01/2003* | *Positive* | *Long-3'UTR* | *Negative* |
| *Ng 03/13* | *01/2003* | *Positive* | *Short-3'UTR* | *Negative* |
| *Ng 03/14* | *01/2003* | *-* | *-* | *-* |

**Table S4. Progress of TMGMV infection in rows of *N. tabacum* cv. Samsun plants.** Data indicate the last plant position in the row (1,2, …n) that is infected at each dpi in each of four replicated rows.

| ***Dpi*** | ***96/5***  **TMGMV** | ***99/16* TMGMV** | ***94/5* TMGMV+STMV** |
| --- | --- | --- | --- |
| ***4*** | *1,1,1,1* | *1,2,1,1* | *2,2,2,1* |
| ***7*** | *3,2,2,3* | *3,3,3,3* | *3,3,2,3* |
| ***10*** | *3,3,4,3* | *3,3,4,3* | *3,3,2,3* |
| ***13*** | *3,3,6,4* | *6,5,4,3* | *3,3,2,3* |
| ***17*** | *3,3,6,7* | *7,5,4,4* | *3,3,2,3* |
| ***20*** | *3,3,6,7* | *7,7,4,4* | *3,3,2,3* |
| ***24*** | *3,3,6,7* | *7,7,4,4* | *3,3,2,3* |
| ***27*** | *3,3,6,7* | *7,7,4,4* | *3,3,2,3* |
| ***31*** | *3,3,6,7* | *7,7,4,4* | *3,3,2,3* |

**Table S5. Accumulation of TMGMV in the inoculated plant of each row (first plant) at the inoculated and systemically infected upper leaves at seven and twelve dpi**. Data are shown as mean ng TMGMV RNA / μg total RNA and standard error over the different numbers of infected plants per treatment.

| ***Isolate*** | ***7 dpi inoculated leaves*** | ***7 dpi upper leaves*** | ***12 dpi upper leaves*** |
| --- | --- | --- | --- |
| ***96/5 TMGMV-Long-3’UTR*** | *1693.333 ± 149.479* | *1917.490 ± 299.176* | *1121.25 ± 101.620* |
| ***99/16 TMGMV-Short-3’UTR*** | *139.048 ± 80.978* | *449.75 ± 36.438* | *1487.50 ± 18.908* |
| ***94/5 TMGMV + STMV*** | *5.844 ± 5.685* | *1707.5 ± 281.732* | *175.990 ± 133.627* |
